# Registration and reporting characteristics of trials investigating exercise therapy following total knee arthroplasty: a systematic review

**DOI:** 10.2340/17453674.2026.46047

**Published:** 2026-06-22

**Authors:** Birk M GRØNFELDT, Rasmus S HUSTED, Rasmus H BRØDSGAARD, Line HOLST, Thomas KALLEMOSE, Cathy M CHAPPLE, Carsten B JUHL, Thomas BANDHOLM

**Affiliations:** 1 Department of Clinical Research, Copenhagen University Hospital Amager-Hvidovre, Hvidovre, Denmark; 2 Physical Medicine & Rehabilitation Research-Copenhagen, Department of Physical and Occupational Therapy, Copenhagen University Hospital Amager-Hvidovre, Hvidovre, Denmark; 3 Department of Health Science and Technology, Faculty of Medicine, Aalborg University, Aalborg, Denmark; 4 Centre for Health, Activity and Rehabilitation Research (CHARR), School of Physiotherapy, University of Otago, Dunedin, Otago, New Zealand; 5 Department of Sport Science and Clinical Biomechanics, University of Southern Denmark, Odense; 6 Department of Physiotherapy and Occupational Therapy, Copenhagen University Hospital, Herlev & Gentofte, Denmark; 7 Department of Clinical Medicine, University of Copenhagen, Copenhagen, Denmark; 8 Department of Orthopaedic Surgery, Copenhagen University Hospital Amager-Hvidovre, Hvidovre, Denmark

## Abstract

**Background and purpose:**

Prospectively registering the primary trial outcome is important to reduce selective outcome reporting and increase the trustworthiness of findings, which guide clinical practice. The objectives of our systematic review were to explore and compare the reporting characteristics of prospectively and non-prospectively registered trials investigating exercise therapy following total knee arthroplasty.

**Methods:**

Randomized trials comparing effects of exercise therapy after total knee arthroplasty for osteoarthritis were identified in 4 databases from 2000 to August 12, 2024. One primary outcome per trial was extracted, using a pre-specified hierarchical algorithm, irrespective of outcome domain. Pooled standardized mean differences (SMDs) were calculated on pre-specified outcome domains, and risk-of-bias assessed using the Cochrane Risk-of-Bias tool v2.

**Results:**

94 trials, comprising 9,396 participants, were included, of which 13 were prospectively registered, 33 retrospectively registered, and 48 unregistered. A single primary outcome was defined in 44% of the 94 trials, and 4 trials reported a primary outcome consistent with a prospective registration. The pooled SMD of primary outcomes was 0.06 (95% confidence interval [CI] −0.03 to 0.16) for prospectively registered trials, 0.67 (CI 0.22–1.11) for retrospectively registered trials, and 0.59 (CI 0.32–0.86) for unregistered trials. Lower risk-of-bias ratings and higher proportions of intention-to-treat adherence, dropout reporting, and adverse event reporting were observed among prospectively registered trials.

**Conclusion:**

Among prospectively registered trials we showed smaller effect size estimates between interventions with lower risk-of-bias ratings, and higher proportions of intention-to-treat adherence, dropout reporting, and adverse event reporting in contrast to trials without prospective registration; furthermore, clear specification of a single primary outcome was uncommon among trials evaluating exercise therapy after total knee arthroplasty.

Numerous recent systematic reviews have evaluated exercise therapy interventions following total knee arthroplasty (TKA) [1-10], yet many of these reviews identify limitations due to trial heterogeneity, particularly in intervention content, outcome selection, and reporting practices [2-4,6,7,9,10].

Randomized controlled trials (RCTs) underpin clinical guideline recommendations because they are considered the gold standard for estimating causal intervention effects when randomization, pre-specified analysis plans, and prospective registration are in place [11-13]. Prospective trial registration involves registering details of a trial’s objectives, interventions, and outcomes before enrolment of the first participant [14] and is required by organizations and medical journal editors [15-18]. Prospective registration, including specification of a primary outcome [19], is intended to limit selective outcome reporting and other questionable research practices [20,21]. Despite these requirements for prospective registration [15-17], selective outcome reporting is common [22,23], even in trials published in medical journals considered high-impact [24]. A 2016 meta-epidemiological analysis of 64 Cochrane reviews found unregistered or retrospectively registered trials reporting larger treatment effect estimates than prospectively registered trials [25]. Clinical practice guidelines, and by extension routine treatment decisions, are informed by evidence from RCTs. Hence, a descriptive examination of trial registration characteristics and a quantitative assessment of differences in reported effect sizes by registration status are warranted.

Our review aimed to (i) descriptively explore trial registration characteristics (primary) and (ii) quantitatively assess differences in reported effect sizes (secondary) among prospectively registered and non-prospectively registered trials comparing exercise therapy interventions following TKA.

## Methods

### Eligibility criteria

**Figure 1:**
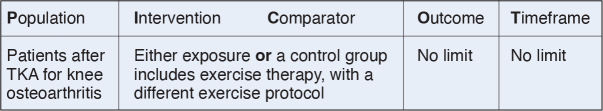
PICOT table. Description of components relating to Population, Intervention, Comparator, Outcome, and Timeframe of interest. TKA: total knee arthroplasty.

We used the PICO(T) framework to define eligibility for the review (Figure 1). Participants were human adults who had undergone TKA followed by an exercise therapy intervention. This may have been the active intervention under investigation, or additional to the active intervention if the effects of exercise were reported separately. Comparators were no exercise, or a type of exercise clearly different from the active group. Any trial outcomes or durations were acceptable for inclusion for the primary aim. Eligible studies were RCTs published after 2000 (launch of clinicaltrials.org) with an accessible full-text publication. Exclusion criteria were studies of TKA for reasons other than osteoarthritis, any RCT with multiple published reports of the same dataset, and any report labelled “secondary analyses” to avoid double-counting participants. Trials were grouped based on whether the registration was prospective, retrospective, or unregistered.

### Information sources

Medline, EMBASE, CINAHL, and the Cochrane Central Register of Randomized Controlled Trials (CENTRAL) were last searched on August 12, 2024. Reference lists of included trials and relevant systematic reviews (published within the last 5 years) were checked for eligible studies and forward citation tracking carried out through Web of Science. No data was sought from other sources mentioned in the PRISMA checklist (e.g., registries). Any inaccessible reports were sought from corresponding authors via mail or researchgate.net.

### Search strategy

The search strategy targeted trial reports of knee arthroplasty and exercise therapy. The Cochrane highly sensitive search strings (adapted to not target drug therapy or animal studies) were applied to primarily identify RCTs in MEDLINE and EMBASE databases (Cochrane Handbook 6.5 chapter 4.4.7). No limits were applied with built-in filters. Search strings are available in Appendix 2.

### Selection process

Before screening, all search results were combined and duplicates removed using the Covidence.org automated duplicate screener and manually by 2 individual reviewers who crosschecked remaining trials as well as group values collected for participant data (BMI, age, n, sex distribution, etc.). Two reviewers independently assessed each record by title and abstract initially, followed by full-text screening. Reviewer disagreements were discussed until consensus. We used Google Translate and DeepL.com to translate trials in languages unfamiliar to reviewers. There was no further use of automation tools, AI, or crowdsourcing for eligibility screening.

### Data extraction

Data extraction involved multiple authors, with each study reviewed by a pair of authors independently. Disagreements were discussed until consensus. No missing data was sought from authors of included trials as we wished to synthesize publicly available data. Data points only available from figures were extracted and quantified using plotdigitizer.com.

### Data items (outcomes)

**Figure 2:**
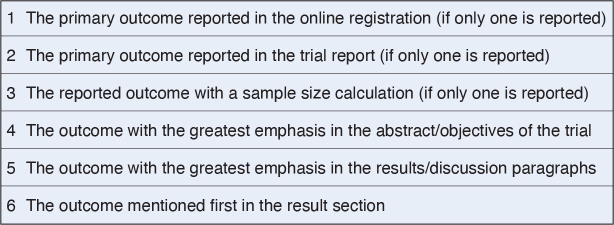
Primary outcome hierarchy. Description of the hierarchy used to select a primary outcome for inclusion. Selection would start at level 1 and if conditions were not met then level 2, checking the level below, and so forth. For each included trial, a single outcome was selected for quantitative synthesis using this prespecified hierarchical decision rule. First, the single primary outcome explicitly specified in the trial’s online registration was selected. If multiple or no primary outcomes were registered, the single clearly stated primary outcome reported in the trial publication was selected. Again, if multiple or no primary outcomes were stated, the outcome used for sample size calculation was selected, provided it was uniquely identifiable. If not, the outcome most emphasized in the abstract or objectives or, when ambiguity remained, the one most emphasized in the results or discussion sections was selected. Ultimately, when no other criteria were met, the first outcome reported in the results section was selected.

Data extracted included: trial design (registration, multicenter, number of study arms), publication details (author, year, location), population (age, sex, BMI, type of surgery), intervention and control details (description, initiation, timing, duration, differentiation), and reporting completeness (sample size calculation, adherence to intention-to-treat principles [ITT], adverse events, dropouts). For each included trial, a single outcome was selected for quantitative synthesis using a prespecified hierarchical decision rule (Figure 2). This was done to prioritize protocol-level registration data, support transparent reporting, and reduce the risk of post hoc outcome selection (see details on changes to the selected outcome in Appendix 9 under 1st amendment). Primary outcome selection was carried out independently by 2 authors. Disagreements were discussed till consensus. Data for primary endpoints was extracted (mean and standard deviation [SD] at baseline and follow-up), in the same way by 2 authors and discussed till consensus. Details of each extracted variable are given in the key table available in the data repository available through the osf.io registration [dataset] [26].

For trials with defined primary outcomes not within the domains of pain, disability, performance-based function, or a combination thereof, the primary outcome score was not extracted. In studies with multiple outcome assessment timepoints (baseline and/or follow-up) or with both intention-to-treat and naive analyses, the one specified for the trial’s primary analysis was chosen (if available). When these were not specified after using the hierarchy (see Figure 2), the following extraction rules were used. If not specified, baseline was always the timepoint closest to initiation of intervention, and the follow-up timepoint, if not specified, was always the one closest to the completion of intervention. Intention-to-treat analysis outcomes were prioritized, unless another analysis was clearly specified as the primary outcome. We used conversion methods for trials reporting standard errors (SE) or 95% confidence intervals (CI) with no SDs and small sample size correction for trials with fewer than 60 participants (Chapter 6.5.2.2 in the Cochrane Handbook 6.5) [27].

### Study risk of bias assessment

Risk of bias of the primary outcome was assessed for the effect of assignment to the intervention (ITT) using the Cochrane Collaborations Risk of Bias 2 tool (RoB2) [28]. The RoB2 assessment was deemed adequate to assess both baseline and follow-up values (i.e., the comparison between the 2). Each study was assessed independently by 2 assessors and disagreements were discussed until consensus.

### Effect measures and covariates

To evaluate the influence of registration status on primary outcomes, continuous data (means and SDs) extracted from available outcome measures was used for statistical analyses. Study characteristics and the RoB2 evaluation are reported according to registration status. Analysis was performed evaluating registration status and the study characteristics listed in Table 1, excluding age, sex, BMI, and geographical location.

**Table 1:** Trial reporting characteristics

Item	Prospective (n = 13)	Retrospective (n = 33)	Unregistered (n = 48)	Total (n = 94)
Demographics				
Age, median (IQR)	66.4 (64.7–68.0)	68.4 (66.9–70.9)	68.7 (67.3–69.9)	68.5 (66.6–70.0)
Sex, n (%)				
Female	1,348 (59)	1,744 (62)	3,246 (76)	6,378 (68)
Male	932 (41)	1,050 (38)	1,036 (24)	3,018 (32)
Body mass index, median (IQR)	32.1 (30.9–33.4)	29.9 (28.4–31.2)	30.1 (28.1–31.5)	30.3 (28.4–31.8)
Participants in trial, median (IQR) (range)	105 (60–334) (30–422)	60 (40–108) (12–290)	49 (35–91) (10–345)	60 (38.5–109.5) (10–422)
Geographic location, n by country	AU = 5	US = 6	US = 7	US = 14
	TW, UK = 2	AU = 4	DE = 7	AU = 10
	FI, KR, US, DK = 1	FI = 3	TR = 5	DE = 9
		KR, CA, TR, DE, CN,	KR, TW, CA = 3	TR = 7
		JP, NO, ES, DK = 2	CN, JP, PK, AT, IR,	KR = 6
		IT, GR = 1	FR = 2	TW, FI, CA = 5
			IT, GR, PL, BR, UK,	CN, JP = 4
			NL, AU, FI = 1	UK, DK = 3
				PK, AT, IR, FR, IT,
				NO, ES, GR = 2
				PL, BR, NL = 1
Design				
Multicenter study (Y/N), n	7/6	11/22	5/43	23/71
Single primary outcome definition (Y/N), n	8/5	18/15	15/33	41/53
Study arms (4/3/2 trial arms), n	0/2/11	1/4/28	0/4/44	1/10/83
ROB 2 assessment summary, n				
1. Bias arising from the randomization process (high/some concerns/low)	1/1/11	1/8/24	9/14/25	11/23/60
2. Bias due to deviations from intended interventions (high/some concerns/low)	1/5/7	11/16/6	23/21/4	35/42/17
3. Bias due to missing outcome data (high/some concerns/low)	2/2/9	6/5/22	9/16/23	17/23/54
4. Bias in measurement of the outcome (high/some concerns/low)	0/4/9	7/12/14	18/18/12	25/34/35
5. Bias in selection of the reported result (high/some concerns/low)	4/4/5	14/17/2	23/25/0	41/46/7
Overall bias (high/some concerns/low)	5/5/3	22/10/1	39/9/0	66/24/4
Reporting				
Primary outcome extracted from, n				
1: Online registration	7	13	0	20
2: Trial report	3	6	11	20
3: Sample size calculation	2	5	7	14
4: Emphasis in abstract/objectives	0	3	10	13
5: Emphasis in results/discussion	0	0	4	4
6: First outcome reported in results	1	6	16	23
Sample size calculations reported (Y/N) **^b^**, n	12/1	28/5	23/25	63/31
Dropouts reporting (yes/partial/no), n	13/0/0	27/2/4	34/8/6	74/10/10
Adverse events reporting (yes/partial/no), n	8/0/5	17/3/13	22/2/24	47/5/42
Data analysis adherent to intention-to-treat, n principles (yes/no/unclear)	8/5/0	10/14/910/31/7	28/50/16	
Interventions				
Intervention initiation timing, median (IQR) in days after surgery	21 (3.5–42.1)	11 (3.4–40.5)	7.2 (1.0–16.5)	8.0 (2.0–31.5)
		1 NA	8 unclear	1 NA, 8 unclear
“Inactive” comparator, n	2	1	5	8
Baseline measurement timing, n (preop./postop./unclear)	5/6/2	16/14/3	21/26/1	42/46/6
Follow-up timepoint, days, median (IQR)	180 (104.5–365)	70 (38.5–105)	60 (28–91)	70 (33.8–127.5)
Days from surgery (extracted outcome) **^a^**	1 NA	2 NA	3 NA	6 NA

Presentation of trial population, design, risk-of-bias, and intervention characteristics. Intention-to-treat definition: all randomized participants clearly included in assigned groups for analysis. NA = not available

a Extracted either at primary outcome timepoint if specified, otherwise selected directly following end of planned exercise therapy intervention.

b Post-hoc calculations are counted as sample size not being reported

### Statistics

The statistical analysis plan (SAP) was made publicly available on March 6, 2025 (after latest search, before any analysis) at https://osf.io/kwqcb/files/osfstorage. For analysis, the primary outcomes across the following domains were combined: self-reported pain, disability, performance-based function, and composites of these domains (such as total scores from WOMAC or KOOS questionnaires). The primary outcomes were pooled to form the analytical estimand (pooled SMD) for the statistical analyses of the current review, rather than a prespecified clinical outcome domain. The effect of exercise therapy compared with the comparator is expressed as the Hedges’ g standardized mean difference (SMD), a bias corrected adjustment of Cohens d, suggested by the Cochrane Collaboration, and analyzed by meta-analysis using random effect modelling (Restricted Maximum Likelihood [REML]). SMD was applied to enable comparison across trials as the focus was on relative effect sizes rather than clinical interpretation per domain. Meta-regression analysis was used to estimate the effect of registration status on the pooled SMD, as well as the effect of the secondary covariates. Additionally, possible effect modification from the study characteristics on registration status was analyzed by extending the meta-regressions to include an interaction term with registration status. All models were adjusted for baseline estimates. All extracted outcomes were “score inverted/normalized” so higher scores indicated improvement/benefit). The overall pooled effects (SMD) were presented so positive outcomes are in favor of the intervention group. Differences in effect between studies were expressed as inconsistency (I^2^) as proposed by Higgins et al. (i.e., percentage of total variation across studies due to heterogeneity, rather than chance) [29]. The magnitudes of the effect sizes were interpreted using the guidelines from Cohen (0.2 = small, 0.5 = moderate, and 0.8 = large) [30]. The impact of the secondary covariates in the meta regression was evaluated by their ability to reduce the between study variance, rather than chance, expressed as tau^2^. All P values from analysis of the secondary variables were presented with correction for multiple testing by Bonferroni correction, with P values multiplied by the number of secondary variables. No missing data was imputed.

### Sensitivity analysis

The ICJME formulated specific requirements for prospective registration of trials for medical journals in 2004 [17]. As it might not be reasonable to include studies before this requirement was published, a sensitivity analysis (not pre-specified in the protocol) was performed, using only studies after July 1, 2005 (end of the ICMJE grace period). All sensitivity analyses were performed as described in the statistics section.

### Ethics, registrations, data sharing plan, use of AI, funding, and disclosures

Ethical approval is not required for this study, as it is based solely on analyses of publicly available, aggregated data from trials and registrations. This prospectively registered systematic review (October 25, 2019, osf.io/6wzre) is reported in accordance with PRISMA 2020 [31] (Appendix 1). The protocol (October 4, 2019), Statistical Analysis Plan (March 6, 2025), data, and code, are publicly available: https://osf.io/kwqcb/files/osfstorage [dataset] [26] under the license CC-BY 4.0 International. None of the authors declare any financial or personal conflicts of interest. This work was not commissioned; no funding was received specifically for its conduct. AI was used for grammar and spelling and the output was controlled by the main author. Complete disclosure of interest forms according to ICMJE are available on the article page, doi: 10.2340/17453674.2026.46047

## Results

**Figure 3:**
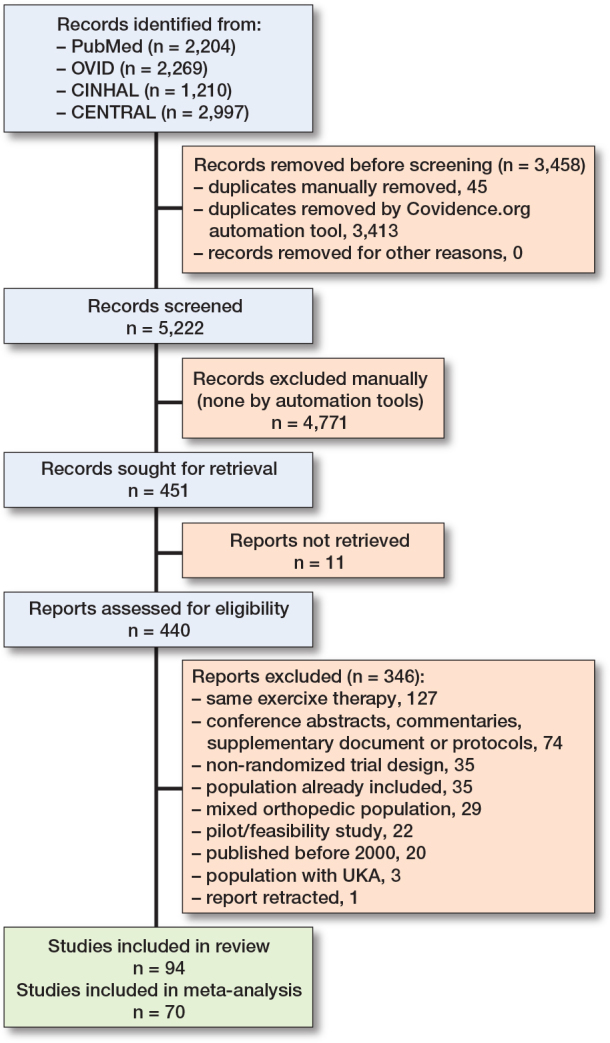
PRISMA flow diagram. Presentation of the process of identifying, screening, and including trials. Reports excluded are cited in Appendix 3

**Figure 4:**
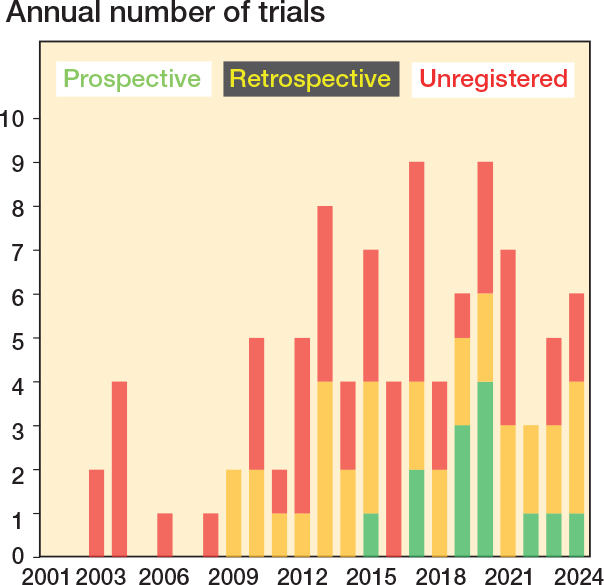
Distribution of included trials over time. X-axis = publication year, Y-axis = number of published trials.

A total of 8,680 records were identified through database searches (Figure 3). After duplicates were removed, 5,222 records were screened, 440 full-text documents assessed, and 94 (13 prospective, 33 retrospective, 48 unregistered) reports were included for the main analysis (Table 1), comprising 9,396 participants (Figure 4—references in Appendix 10). Data from 70 (9 prospective, 25 retrospective, 36 unregistered) reports were included in secondary analyses (forest plot and meta-regression analyses—Figure 6 and Appendices 6 and 7). Reasons for exclusion by full-text screening were most often that interventions compared the same exercise therapy, or were conference abstracts, commentaries, etc. (i.e., not full trial reports) (Appendix 3). No additional reports were found by searching references or cited-by lists.

### Trial design differences

Included trials with prospective registration presented with a higher median of participants, longer time until follow-up (for the extracted primary outcome), and more frequently used multicenter designs than non-prospectively registered trials (see Table 1). For a more detailed overview of each trial, see the raw data extraction sheet and risk of bias assessments available in the data repository available through the registration [dataset] [26].

A brief description of the intervention and comparator groups, the selected outcome, and risk of bias domain judgments is available for all 94 included trials in Appendix 4.

### Trial reporting differences

Across reporting domains (see Table 1), the proportion of trials meeting reporting criteria was consistently highest among prospectively registered trials, intermediate among retrospectively registered trials, and lowest among unregistered trials. This pattern was observed for: definition of a single primary outcome (62%/55%/31%), reporting of sample size calculations (92%/85%/48%), dropout reporting (100%/82%/71%), adverse event reporting (62%/52%/46%), and adherence to intention-to-treat principles (62%/30%/21%) (numerators and denominators available in Table 1).

In the 13 prospectively registered trials,: a single primary outcome consistent with a single prospectively registered, unaltered, primary outcome was reported in 4 of the 13 trials. The primary outcome was changed or switched in 2 of the 13 trials. Multiple primary outcomes were registered and/or reported in 7 of the 13 trials (see Appendix 10 for references).

### Risk of bias in trials

**Figure 5:**
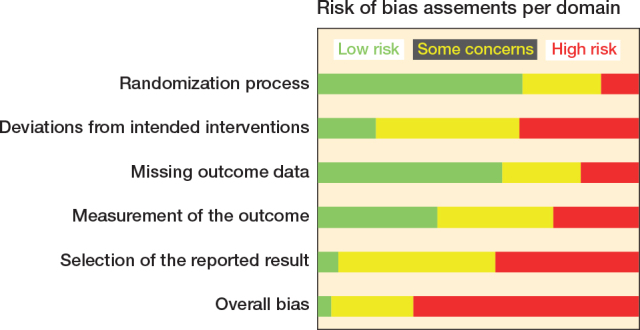
Risk of bias domains. Summary of risk of bias assessments for the included primary outcome using the Cochrane Risk of Bias 2 (RoB2) tool. N = 94.

Outcomes in 4 of 94 (4.3%) trials were judged at low risk of bias, 24 of 94 (26%) at some concern of bias, and 66 of 94 (70%) at high risk of bias (Figure 5). Prospectively registered trials represented up to 3 of the 4 trial outcomes judged at low risk of bias, 5 of the 24 (21%) at some concerns for risk bias, and 5 of the 66 (7.6%) at high risk of bias. An overview of the risk of bias is available in Table 1, and an extended risk of bias table presenting experimental and control exercise therapy interventions is available in Appendix 4. The complete raw assessments are available in the data repository [dataset] [26].

### Study effect differences

**Figure 6:**
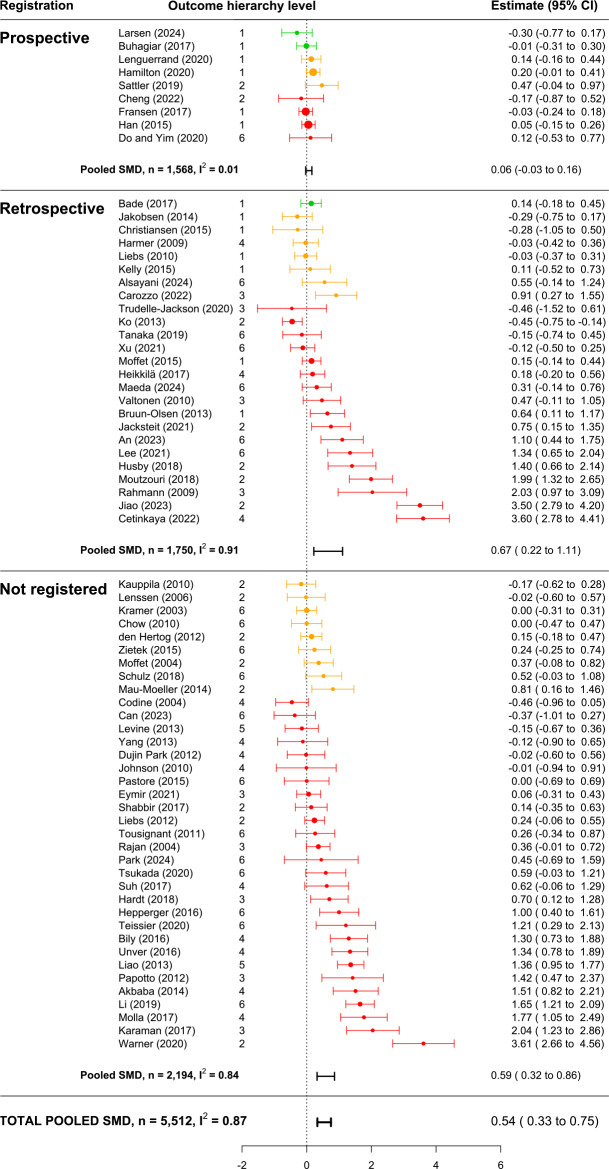
Forest plot. Presentation of pooled standardized mean differences of prospective (n = 9), retrospective (n = 25), and unregistered (n = 36) RCTs with extractable trial data within the domains of pain, disability, performance-based function, or a combination of those 3. A standardized mean difference favoring the exposure group (the group with more exercise) is represented with values above 0, values below 0 favor the control group (the group with less exercise). Green represents outcomes judged at low risk of bias, yellow outcomes judged at some concerns for bias, and red outcomes judged at high risk of bias. I^2^ represents total variation across studies due to heterogeneity, rather than chance.

The pooled SMD of prospectively registered trials was 0.06 (CI –0.03 to 0.16), not favoring either group, whereas the pooled SMD of retrospectively trials was 0.67 (CI 0.22–1.11] and unregistered trials 0.59 (CI 0.32–0.86), both favoring the intervention group (Figure 6). Very low I^2^ heterogeneity was present between prospectively registered trials, and high I^2^ heterogeneity presented for the retrospectively registered and unregistered trials. Funnel plots are available in Appendix 5.

The meta-regression analysis (Appendix 6) of the included trials observed the following differences: in retrospectively and unregistered trials the pooled SMD was 0.53 (CI –0.03 to 1.11) and 0.37 (CI –0.19 to 0.92) larger than for the prospectively registered trials. The covariates: outcome hierarchy levels, risk of bias, ITT adherence, dropout reporting, and primary outcome definition showed some association with the reported effect size estimate and explained notable between-study variance (tau^2^). None of the differences for the covariates, except ITT adherence, were significant and generally presented large confidence intervals.

The interaction meta-regression analysis (Appendix 7) showed some estimates with interaction effects, those most pronounced being: description of trial dropouts, primary outcome hierarchy level, and adherence to ITT principles. However, none of the interactions were statistically significant after correction for multiple testing, and confidence intervals were generally wide, likely due to few observations being available within some levels.

### Sensitivity analysis

Excluding trials not required to be prospectively registered, according to the July 1, 2005 ICMJE grace period, did not change the interpretation of outcomes (see Appendix 8). Only minor changes were observed: compared with registered trials, the SMD estimate for non-registered trials favored the intervention group slightly more, with slightly larger confidence intervals and tau^2^ values. Furthermore, the Eggers test in the funnel plot for non-registered trials changed from being statistically significant to showing weak/borderline evidence of asymmetry.

## Discussion

We aimed to explore and compare the reporting characteristics of prospectively and non-prospectively registered trials investigating exercise therapy following total knee arthroplasty and observed that, compared with non-prospectively registered trials, the outcomes of prospectively registered trials had smaller effect size differences between interventions with overall lower risk-of-bias ratings, and higher proportions of intention-to-treat adherence, dropout reporting, and adverse event reporting.

The core observation, that non-prospectively registered trials were associated with larger effect sizes than prospectively registered ones, mirrors the finding of the previously referred meta-epidemiological study of Cochrane reviews [25]. Fewer than half of the included trials clearly defined a single primary outcome, and among the 13 trials with pre-registered outcomes, but 4 of the 13 consistently reported the same preregistered single primary outcome in the final report (Appendix 10). Riley et al. found 49 of 138 (36%) trials on musculoskeletal physical therapy were prospectively registered [32], compared with 13 of 94 (14%) in the current review; this is likely a result of older trials being included in the current review. Riley et al. also found that among prospectively registered trials, 42 of 49 (86%) reported outcomes consistent with registered intent, but post-randomization bias could not be ruled out for the remaining 96 trials [32]. Changing the pre-registered primary outcome after a trial begins is not recommendable, but can happen. It is less of concern if it happens shortly after the trial begins, when little data has been collected, and no analyses could be performed—provided the change is transparently reported. This was the reason why 1 retrospectively registered trial was considered at low risk of bias, as seen in Figure 6. Many of the included trials listed multiple “primary” outcomes without the accompanying methodological specifications, which are often recommended when more than 1 primary endpoint is intended (e.g., an explicit success criterion such as co-primary vs at least 1 prespecified multiplicity control, and sample-size planning aligned with that choice) [33]. Our observation is not intended as a critique of legitimate co-primary or dual-primary frameworks per se, but rather of unclear pre-specification in trials that have labelled several outcomes as “primary.” Such practice may complicate consistent outcome reporting and interpretation and could be associated with variable or larger effect estimates when combined with retrospective or absent registration. Considering the differences in effect sizes between prospectively registered and non-prospectively registered trials in the current sample (see Figure 6), and that just 3 of 94 trials reported a statistical analysis plan, post-randomization bias cannot be excluded in the current sample of RCTs comparing exercise therapy interventions after total knee replacement. While the current review illustrates how timely outcome registration enables readers and peer-reviewers to assess coherence between registration and reporting, timely registration alone does not necessarily ensure higher methodological quality, as that also depends on adherence to other core principles of RCT design and conduct.

A 2023 synthesis of meta-epidemiological studies of rehabilitation trials concluded that inadequate or unclear randomization sequence generation and allocation concealment were likely to produce exaggerated effect estimates for continuous outcomes, and higher risk of reporting bias appeared to be associated with an overestimation of effects [34]. In the current review, most of the extracted outcomes were rated at low risk of bias in the “randomization process” domain, indicating that the differences in effect size estimates were not clearly attributable to randomization and allocation concealment biases. In contrast, the “reporting bias” domain presented with the highest proportion of outcomes at some concern or high risk of bias in retrospectively registered and unregistered trials. This pattern may be consistent with an association between higher risk of reporting bias and larger pooled SMD estimates in those trials.

### Limitations

The statistical analysis plan was registered after completion of data extraction but prior to any analysis. Owing to the approach chosen to select the primary outcome from each trial, the pooled estimates presented in the current meta-analyses do not represent the overall clinical effect of exercise therapy after TKA. Rather, they reflect pooled standardized mean differences based on trial-specific primary outcomes selected using a prespecified hierarchy, across different outcome domains. Consequently, the analyses aggregate outcomes representing different constructs of pain, disability, and performance-based function. While standardized mean differences are typically recommended for combining results within the same construct (Cochrane handbook 6.5, section 15.5.3), they were used here to facilitate comparison of effect size estimates across trials with differing primary outcomes. Accordingly, the analyses were intended to examine whether effect size estimates varied by trial characteristics, rather than to inform clinical effectiveness.

Our review findings show that non-prospectively registered RCTs of exercise therapy interventions after TKA tend to report larger effect size estimates compared with prospectively registered trials. These observations warrant cautious interpretation, as differences in effect size estimates, related to registration practices, may influence evidence synthesis used to inform clinical practice guidelines if not appropriately considered.

### Conclusion

We found that prospective trial registration and clear specification of a single primary outcome were uncommon among trials evaluating exercise therapy after TKA. Prospectively registered trials were observed to differ from retrospectively registered and unregistered trials with respect to larger sample sizes, clearer reporting practices, lower risk-of-bias ratings, and smaller effect size differences between interventions.

### Supplementary data

Appendices 1–10 are available as supplementary data on the article page, doi: 10.2340/17453674.2026.46047

## Supplementary Material





















## Notes

Conception and design: RH, CC, TB, BG, CJ. Trial selection: BG, LH, RB, TB. Collection and assembly of data: BG, TB, CJ, RH, LH, CC, RB. Analysis and interpretation of the data: BG, TB, CJ, TK. Statistical expertise: TK, CJ. Drafting of the article: BG, TB. Critical revision of the article for important intellectual content: TB, CJ, TK, RH, LH, CC, RB. All authors approved the final decision to submit the manuscript for publication.

Handling co-editors: Bart Pijls and Robin Christensen

*Acta* thanks Justine M Naylor and Henricus Vermeulen for help with peer review of this manuscript.
