# Supplementary material for: Registration and reporting characteristics of trials investigating exercise therapy following total knee arthroplasty: a systematic review
Source: Acta Orthop. 2026 Jun 22;97:408–16. doi: 10.2340/17453674.2026.46047 (PMC13284969; doi:10.2340/17453674.2026.46047)
Supplement: Supplementary file 2 [file ActaO-97-46047-s2.pdf]

Cochrane RCT search filters:

Table x The Cochrane Highly Sensitive Search Strategy for identifying randomized trials in MEDLINE, PubMed format	
#1	randomized controlled trial [pt]
#2	controlled clinical trial [pt]
#3	randomized [tiab]
#4	placebo [tiab]
#5	randomly [tiab]
#6	trial [tiab]
#7	groups [tiab]
#8	#1 OR #2 OR #3 OR #4 OR #5 OR #6 OR #7

Table x The Cochrane Highly Sensitive Search Strategy for identifying randomized trials in MEDLINE, Ovid format	
#1	randomized controlled trial.pt.
#2	controlled clinical trial.pt.
#3	randomized.ab.
#4	placebo.ab.
#5	randomly.ab.
#6	trial.ab.
#7	groups.ab.
#8	#1 OR #2 OR #3 OR #4 OR #5 OR #6 OR #7

Pubmed/MEDLINE full search-string:

("randomized controlled trial"[Publication Type] OR "controlled clinical trial"[Publication Type] OR "randomized"[Title/Abstract] OR "placebo"[Title/Abstract] OR "randomly"[Title/Abstract] OR "trial"[Title/Abstract] OR "groups"[Title/Abstract]) AND ("knee arthroplast*"[Title/Abstract] OR "total knee arthroplast*"[Title/Abstract] OR "knee replacement*"[Title/Abstract] OR "total knee replacement*"[Title/Abstract] OR "arthroplasty, replacement, knee"[MeSH Terms]) AND ("exercise"[MeSH Terms] OR "exercise*"[Title/Abstract] OR "Resistance training"[Title/Abstract] OR "Resistance training"[MeSH Terms] OR "exercise therap*"[Title/Abstract] OR "Exercise therapy"[MeSH Terms] OR "rehabilitation*"[Title/Abstract] OR "rehabilitation"[MeSH Terms] OR "physical therap*"[Title/Abstract] OR "Physical therapy modalities"[MeSH Terms] OR "physiotherap*"[Title/Abstract]).

EMBase via OVID

(crossover procedure OR double-blind procedure OR randomized controlled trial OR single-blind procedure).hw. OR (random$ OR factorial$ OR crossover$ OR cross over$ OR placebo$ OR doubl$ blind$ OR singl$ blind$ OR assign$ OR allocate$ OR volunteer$).hw,ab,ti.

(Knee arthroplasty$ OR Total knee arthroplasty$ OR Knee replacement OR Total knee replacement$).ab,ti. OR exp Knee arthroplasty/

(Exercise$ OR Resistance training OR Exercise therap$ OR Rehabilitation$ OR Physical therap$ OR Physiotherap$).ti,ab. OR exp resistance training/ OR exp kinesiotherapy/ OR exp Rehabilitation/ OR exp Physiotherapy/ OR exp exercise/

CiNAHL RCT filter:

Cochrane CiNAHL plus filter, animal trials portion omitted (Glanville et al. 2019)

S1 MH randomized controlled trials

S2 MH double-blind studies

S3 MH single-blind studies

S4 MH random assignment

S5 MH pretest-posttest design

S6 MH cluster sample

S7 TI (randomised OR randomized)

S8 AB (random*)

S9 TI (trial)

S10 MH (sample size) AND AB (assigned OR allocated OR control)

S11 MH (placebos)

S12 PT (randomized controlled trial)

S13 AB (control W5 group)

S14 (MH “crossover design”) OR (MH “comparative studies”)

S15 AB (cluster W3 RCT)

CINAHL via EBSCO

S33	S6 AND S17 AND S32
S32	S18 OR S19 OR S20 OR S21 OR S22 OR S23 OR S24 OR S25 OR S26 OR S27 OR S28 OR S29 OR S30 OR S31
S31	AB (cluster W3 RCT)
S30	(MH “crossover design”) OR (MH “comparative studies”)
S29	AB (control W5 group)
S28	PT randomized controlled trial
S27	(MH "Placebos")
S26	(MH "sample size") AND AB (assigned OR allocated OR control)
S25	TI (trial)
S24	AB (random*)
S23	TI (randomised OR randomized)

S22	(MH "Cluster Sample+")
S21	(MH "Pretest-Posttest Design+")
S20	(MH "Random Assignment")
S19	(MH "Double-Blind Studies") OR (MH "Single-Blind Studies")
S18	(MH "Randomized Controlled Trials+")
S17	S7 OR S8 OR S9 OR S10 OR S11 OR S12 OR S13 OR S14 OR S15 OR S16
S16	TI physical therap* OR AB physical therap* OR TI physiotherap* OR AB physiotherap*
S15	(MH "Physical Therapy+")
S14	TI rehabilitation* OR AB rehabilitation*
S13	(MH "Rehabilitation+")
S12	TI exercise therapy* OR AB exercise therapy*
S11	(MH "Therapeutic Exercise+")
S10	TI resistance training* OR AB resistance training*
S9	(MH "resistance training+")
S8	TI exercise* OR AB exercise*
S7	(MH "exercise+")
S6	S1 OR S2 OR S3 OR S4 OR S5
S5	TI Total knee replacement* OR AB Total knee replacement*
S4	TI Knee replacement* OR AB Knee replacement*
S3	(MH "Arthroplasty, Replacement, Knee+")
S2	TI Total knee arthroplast* OR AB Total knee arthroplast*
S1	TI Knee arthroplast* OR AB Knee arthroplast*

Cochrane Central Register of Randomized Controlled Trials (CENTRAL)

ID	Search	Hits
#1	(Knee arthroplast*):ti,ab,kw	

#2 (Total knee arthroplast*):ti,ab,kw

#3 (Knee replacement?):ti,ab,kw

#4 (Total knee replacement?):ti,ab,kw

#5 MeSH descriptor: [Arthroplasty, Replacement, Knee] explode all trees

#6 #1 OR #2 OR #3 OR #4 OR #5

#7 MeSH descriptor: [Exercise] explode all trees

#8 (exercise? OR exercise*):ti,ab,kw

#9 (resistance training):ti,ab,kw

#10 MeSH descriptor: [Resistance Training] explode all trees

#11 (exercise therap* OR exercise therap?):ti,ab,kw

#12 MeSH descriptor: [Exercise Therapy] explode all trees

#13 (rehabilitation* OR rehabilitation?):ti,ab,kw

#14 MeSH descriptor: [Rehabilitation] explode all trees

#15 (physical therap* OR physical therap?):ti,ab,kw

#16 MeSH descriptor: [Physical Therapy Modalities] explode all trees

#17 (physiotherap* OR physiotherap?):ti,ab,kw

#18 #7 OR #8 OR #9 OR #10 OR #11 OR #12 OR #13 OR #14 OR #15 OR #16 OR #17

#19 #6 AND #18
