# Supplementary figures and images for: Registration and reporting characteristics of trials investigating exercise therapy following total knee arthroplasty: a systematic review
Source: Acta Orthop. 2026 Jun 22;97:408–16. doi: 10.2340/17453674.2026.46047 (PMC13284969; doi:10.2340/17453674.2026.46047)

**Prospective**

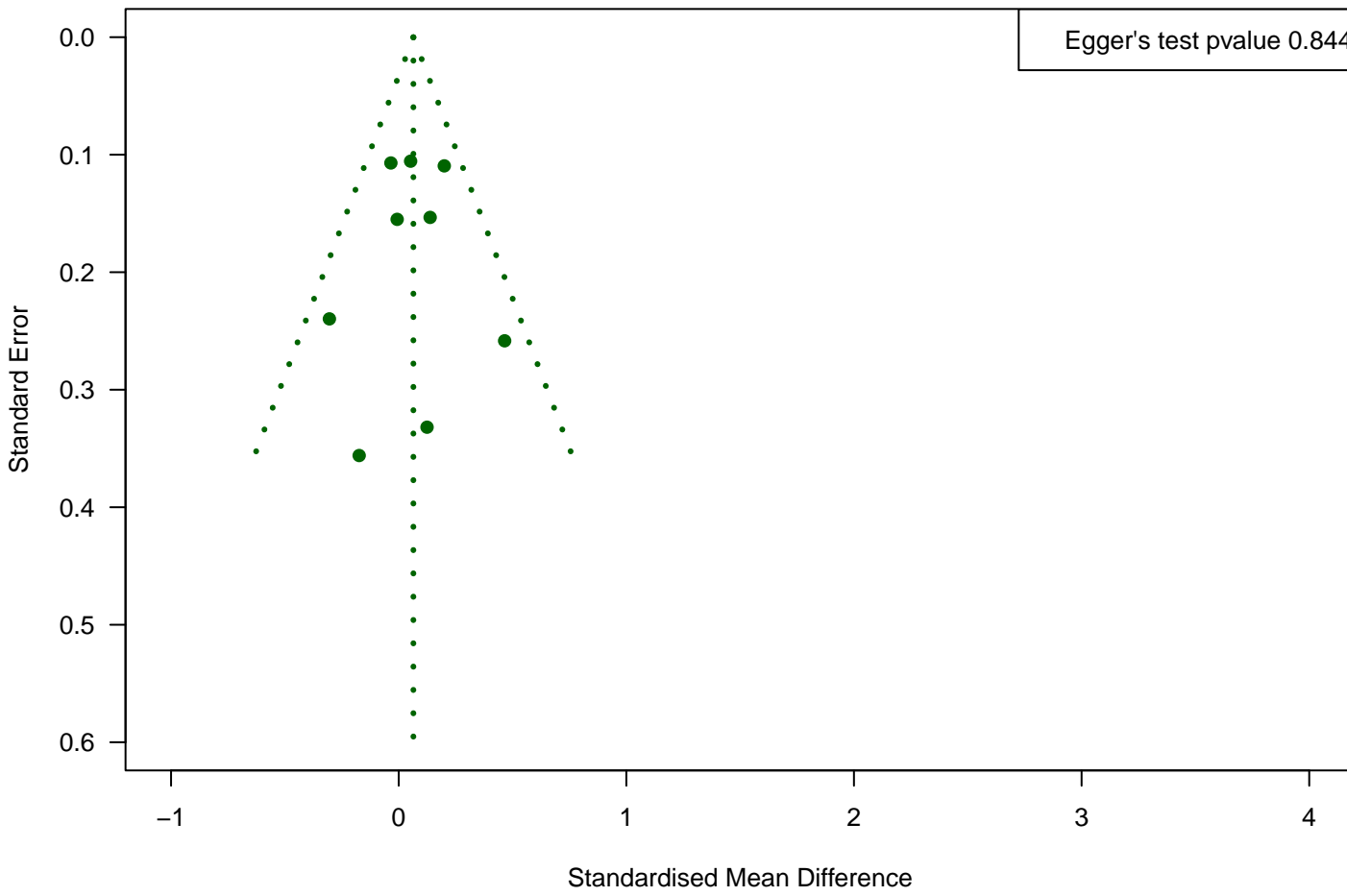

**Not registered**

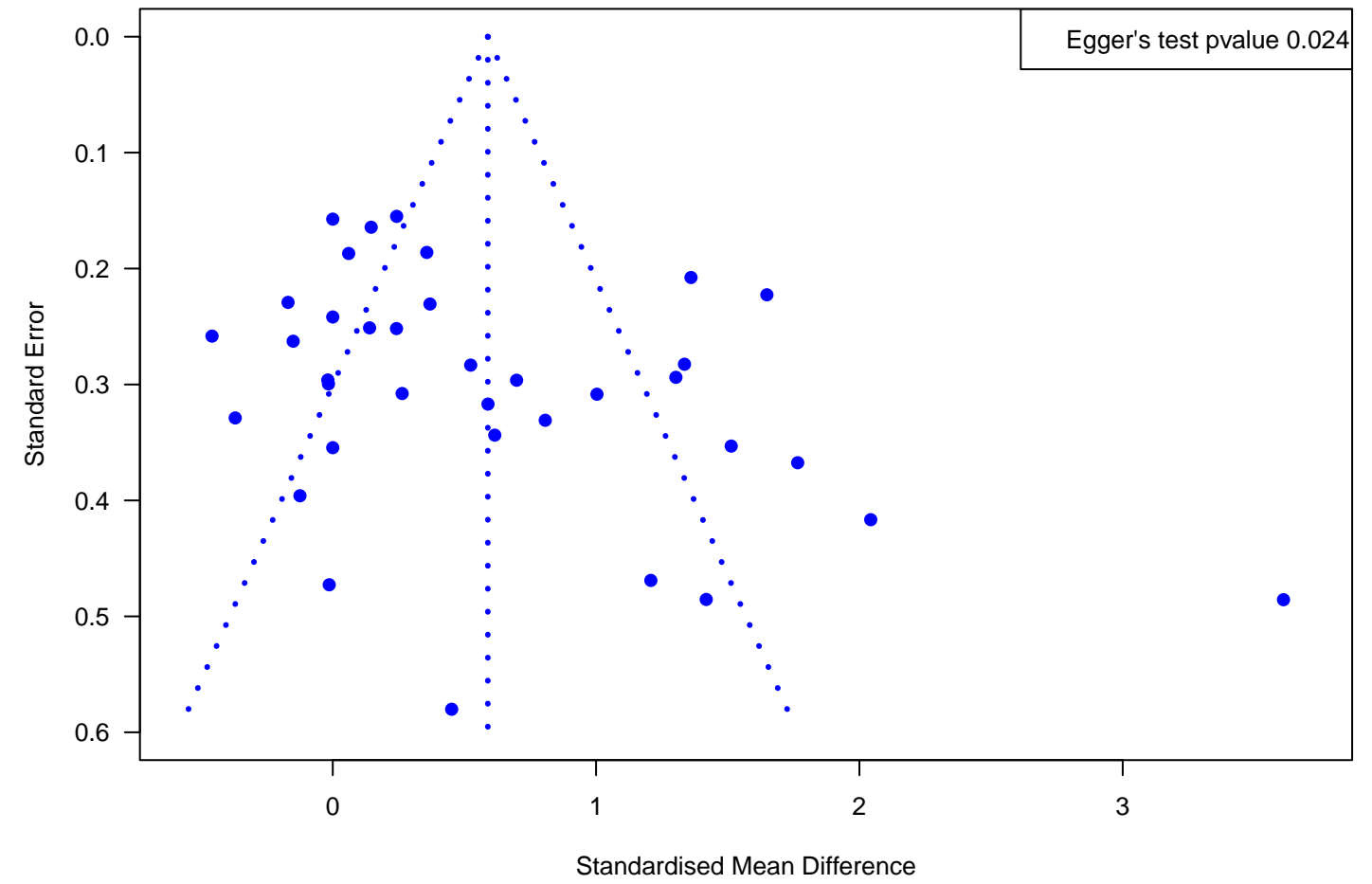

**Retrospective**

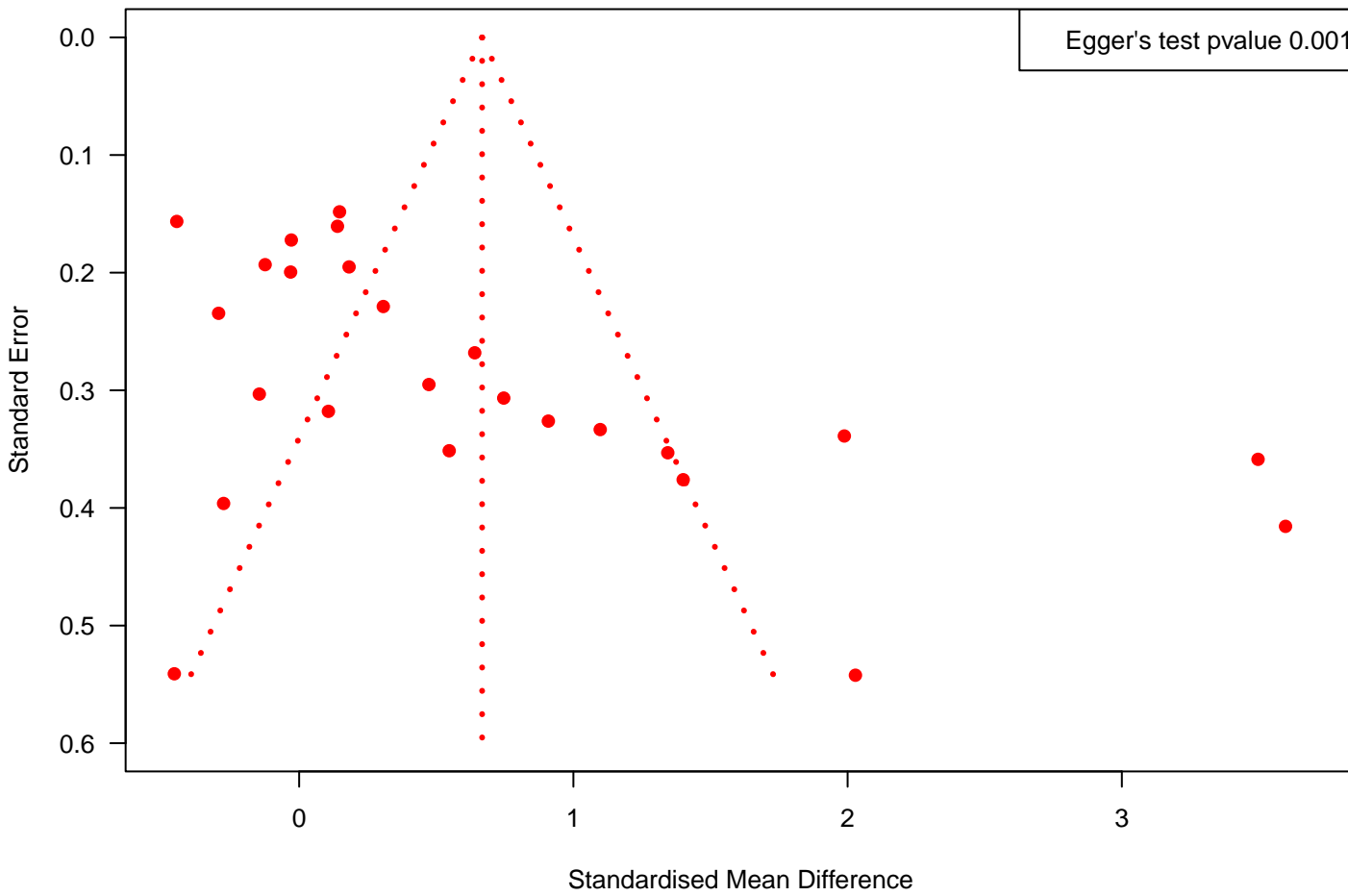

**Combined**

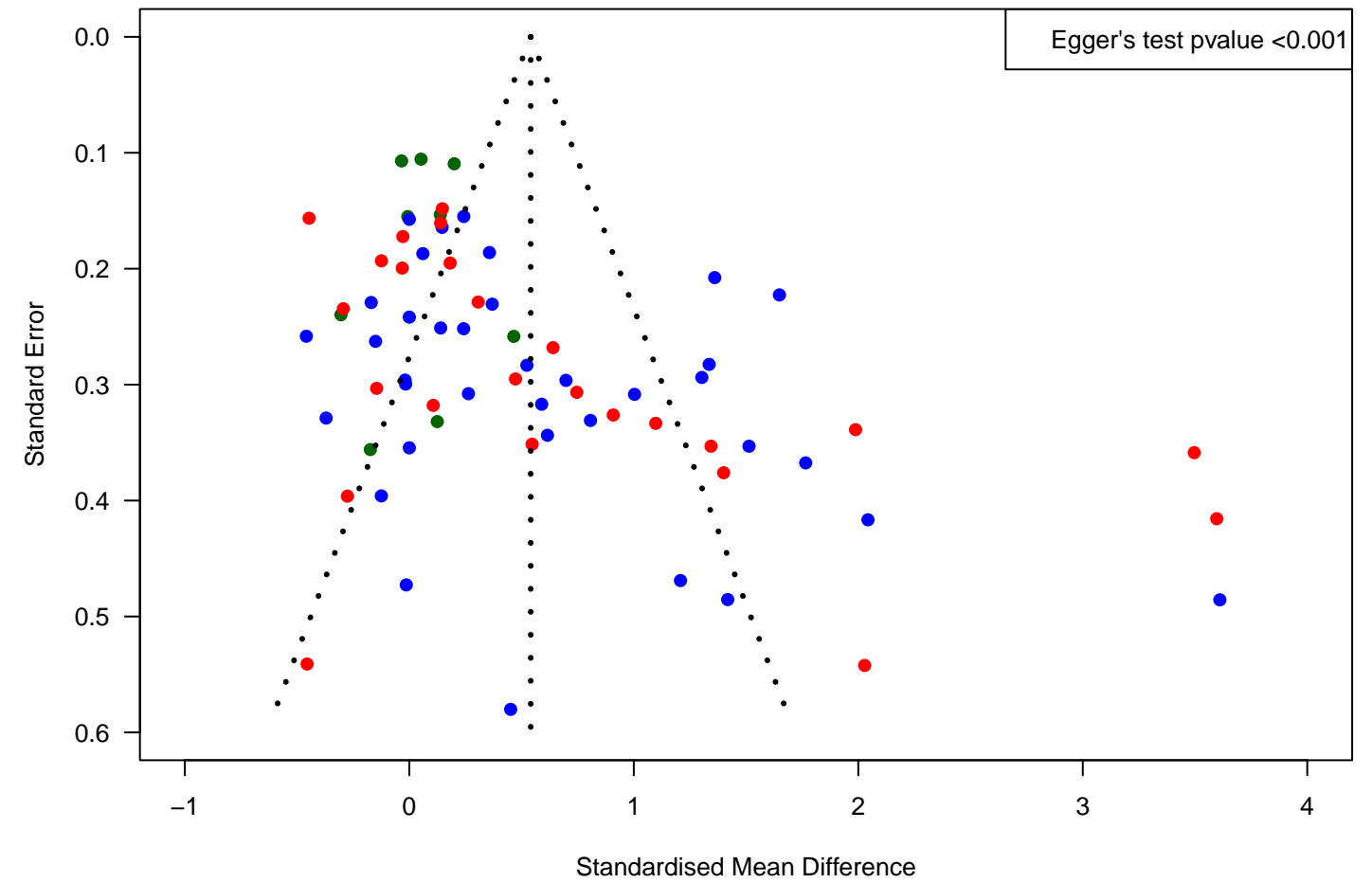

Supplement: Supplementary file 5 [file ActaO-97-46047-s5.pdf]
