# Supplementary material for: Registration and reporting characteristics of trials investigating exercise therapy following total knee arthroplasty: a systematic review
Source: Acta Orthop. 2026 Jun 22;97:408–16. doi: 10.2340/17453674.2026.46047 (PMC13284969; doi:10.2340/17453674.2026.46047)
Supplement: Supplementary file 6 [file ActaO-97-46047-s6.pdf]

Covariate	Comparison	Difference in SMD	95% CI	P-value	Bonferroni-corrected P-value	Tau^2	Tau^2 impact (% reduction of between study variance)	Studies excluded (missing variables)
Registration time	Retrosp. compared to Prosp.	0.53	[-0.03, 1.11]	0.070	-	0.463	27.2%	(90)
	Unreg. compared to Prosp.	0.37	[-0.19, 0.92]	0.190	-			
Participants	Per 10 additional participants	-0.02	[-0.05, 0.01]	0.088	1	0.453	28.8%	(90)
Multicentre study	Yes compared to No	-0.40	[-0.81, 0.01]	0.051	0.763	0.445	30.0%	(90)
Reported a single primary outcome<br>(not hierarchy)	Yes compared to No	-0.14	[-0.52, 0.23]	0.450	1	0.476	25.1%	(90)
Study arms	3 or more compared to 2	-0.23	[-0.82, 0.36]	0.437	1	0.475	25.3%	(90)
Risk of Bias	Low compared to High	-0.60	[-1.45, 0.25]	0.164	1	0.443	30.3%	(90)
	Some concerns compared to High	-0.42	[-0.83, -0.02]	0.040	0.607			
Primary outcome hierarchy level<br>1 – Online registration,<br>2 – Trial report,<br>3 – Sample size calculation,<br>4 – Emphasis in abstract/objectives,<br>5 – Emphasis in results/discussion,<br>6 – First p/d/pbf/composite outcome in results	2 compared to 1	0.50	[-0.08, 1.07]	0.088	1	0.465	26.9%	(90)
	3 compared to 1	0.62	[-0.06, 1.30]	0.072	1			
	4 and 5 compared to 1	0.54	[-0.06, 1.13]	0.076	1			
	6 compared to 1	0.34	[-0.20, 0.88]	0.213	1			
Sample size calculation reported	Yes compared to No	0.13	[-0.25, 0.52]	0.494	1	0.480	24.5%	(90)
Dropouts description	Yes compared to No	-0.62	[-1.27, 0.02]	0.057	0.849	0.457	28.1%	(90)
	Partial compared to No	-0.71	[-1.67, 0.25]	0.143	1			
Adverse events reporting	Yes compared to No	-0.08	[-0.48, 0.32]	0.680	1	0.489	23.1%	(90)
	Partial compared to No	-0.10	[-1.05, 0.86]	0.839	1			
Adhering to intent to treat principles	Not adhering compared to adhering	0.40	[0.03, 0.77]	0.036	0.540	0.368	42.1%	(90)
	Unclear compared to adhering	1.04	[0.49, 1.58]	<0.001	0.005			
Time since surgery<br>Days since surgery to intervention start	Initiated 100 days later	<0.01	[-0.07, 0.09]	0.765	1	0.495	22.1%	(1,16,18,73,90)
Intervention duration<br>Time between initiation and follow-up	Per 100 days longer	-0.17	[-0.37, 0.02]	0.078	1	0.464	27.0%	(1,16,18,73,90)

Covariate	Comparison	Difference in SMD	95% CI	P-value	Bonferroni-corrected P-value	Tau^2	Tau^2 impact (% reduction of between study variance)	Studies excluded (missing variables)
Primary outcome domain	Disability compared to Composite	0.31	[-0.36, 0.98]	0.355	1	0.487	23.4%	(90)
	Pain compared to Composite	0.04	[-0.66, 0.74]	0.907	1			
	PBF compared to Composite	0.03	[-0.57, 0.63]	0.915	1			
Baseline measurement timing	Before surgery compared to after	0.05	[-0.33, 0.44]	0.783	1	0.495	22.1%	(31,50,57,90)
Time to follow-up	Per 100 days longer	-0.01	[-0.08, 0.06]	0.754	1	0.486	23.6%	(16,90)

Appendix 6 Meta regression analysis. Presentation of the influence of secondary covariates on SMD. Registration status was used as primary exposure for the analysis; hence Bonferroni correction was omitted for registration status. Primary outcome hierarchy levels: 1 = online registration, 2 = trial report, 3 = sample size calculation, 4,5 = emphases in abstract/objectives/results/discussion (grouped as there were too few), 6 = first outcome in results. PBF = Performance-based-function. Includes the 70 trials reporting extractable and comparable primary outcome measures, trials omitted from the analysis are referenced. Raw Tau^2 = 0.6358. References for excluded studies available in Appendix 10.
