# Supplementary material for: Registration and reporting characteristics of trials investigating exercise therapy following total knee arthroplasty: a systematic review
Source: Acta Orthop. 2026 Jun 22;97:408–16. doi: 10.2340/17453674.2026.46047 (PMC13284969; doi:10.2340/17453674.2026.46047)
Supplement: Supplementary file 7 [file ActaO-97-46047-s7.pdf]

Covariate	Comparison	Interaction	95% CI	P-value	Bonferroni-corrected P-value	Tau^2	Tau^2 impact (% reduction of between study variance)	Studies excluded (missing variables)
Participants	Pr. 10 extra participants in study							
	Retrosp. vs. Prosp.	0.063	[-0.01, 0.14]	0.094	1	0.448	29.5%	(90)
	Nonreg. vs. Prosp.	0.015	[-0.06, 0.09]	0.677	1			
Multicentre study	Yes compared to No							
	Retrosp. vs. Prosp.	0.597	[-0.62, 1.82]	0.333	1	0.452	28.9%	(90)
	Nonreg. vs. Prosp.	0.183	[-1.19, 1.56]	0.791	1			
Reported a single primary outcome (not hierarchy)	Yes compared to No							
	Retrosp. vs. Prosp.	0.064	[-1.16, 1.29]	0.918	1	0.489	23.1%	(90)
	Nonreg. vs. Prosp.	0.056	[-1.17, 1.28]	0.927	1			
Study arms	3 or more compared to 2							
	Retrosp. vs. Prosp.	0.412	[-1.17, 1.99]	0.603	1	0.485	23.7%	(90)
	Nonreg. vs. Prosp.	0.299	[-1.25, 1.85]	0.701	1			
Risk of Bias	Low compared to High							
	Retrosp. vs. Prosp.	0.421	[-1.53, 2.38]	0.668	1	0.466	26.7%	(90)
	Nonreg. vs. Prosp.	N/A	N/A	N/A	N/A			
	Some concerns compared to High							
	Retrosp. vs. Prosp.	0.628	[-0.73, 1.99]	0.359	1			
	Nonreg. vs. Prosp.	0.349	[-0.95, 1.65]	0.593	1			
Primary outcome hierarchy level	2 compared to 1							
1 – Online registration,	Retrosp. vs. Prosp.	-1.178	[-2.70, 0.34]	0.126	1	0.475	25.3%	(90)
2 – Trial report,	Nonreg. vs. Prosp.	-0.089	[-2.15, 1.97]	0.931	1			
3 – Sample size calculation,	3 compared to 1							
4 – Emphasis in abstract/objectives,	Retrosp. vs. Prosp.	N/A	N/A	N/A	N/A			
5 – Emphasis in results/discussion,	Nonreg. vs. Prosp.	0.118	[-1.99, 2.22]	0.911	1			
6 – First p/d/pbf/composite outcome in results	4 and 5 compared to 1							
	Retrosp. vs. Prosp.	N/A	N/A	N/A	N/A			
	Nonreg. vs. Prosp.	0.613	[-1.45, 2.68]	0.554	1			
	6 compared to 1							
	Retrosp. vs. Prosp.	-0.119	[-1.99, 1.75]	0.899	1			
	Nonreg. vs. Prosp.	N/A	N/A	N/A	N/A			
Sample size calculation reported	Yes compared to No							
	Retrosp. vs. Prosp.	-0.528	[-2.26, 1.21]	0.546	1	0.480	24.5%	(90)
	Nonreg. vs. Prosp.	-0.067	[-1.68, 1.55]	0.934	1			

Covariate	Comparison	Interaction	95% CI	P-value	Bonferroni-corrected P-value	Tau²	Tau² impact (% reduction of between study variance)	Studies excluded (missing variables)
Dropouts description	Yes compared to No							
	Retrosp. vs. Prosp.	N/A	N/A	N/A	N/A	0.405	36.3%	(90)
	Nonreg. vs. Prosp.	-1.630	[-2.86, -0.40]	0.010	0,157			
	Partial compared to No							
	Retrosp. vs. Prosp.	N/A	N/A	N/A	N/A			
	Nonreg. vs. Prosp.	-2.200	[-4.23, -0.18]	0.033	0,497			
Adverse events reporting	Yes compared to No							
	Retrosp. vs. Prosp.	0.466	[-0.75, 1.68]	0.448	1	0.437	31.3%	(90)
	Nonreg. vs. Prosp.	-0.598	[-1.76, 0.56]	0.305	1			
	Partial compared to No							
	Retrosp. vs. Prosp.	N/A	N/A	N/A	N/A			
	Nonreg. vs. Prosp.	-0.085	[-1.84, 2.01]	0.930	1			
Following intent to treat analysis principles	Per protocol compared to ITT							
	Retrosp. vs. Prosp.	-0.361	[-1.52, 0.80]	0.534	1	0.363	42.9%	(90)
	Nonreg. vs. Prosp.	-0.381	[-1.50, 0.74]	0.498	1			
	Unclear compared to ITT							
	Retrosp. vs. Prosp.	N/A	N/A	N/A	N/A			
	Nonreg. vs. Prosp.	0.998	[-0.15, 2.15]	0.089	1			
Time since surgery (intervention start)	Initiated 100 days later							
	Retrosp. vs. Prosp.	0.139	[-0.24, 0.52]	0.469	1	0.485	23.7%	(1,16,18,73,90)
	Nonreg. vs. Prosp.	-0.073	[-0.25, 0.10]	0.413	1			
Intervention duration (follow-up – initiated)	100 days longer							
	Retrosp. vs. Prosp.	0.272	[-0.22, 0.76]	0.268	1	0.479	24.6%	(1,16,18,73,90)
	Nonreg. vs. Prosp.	0.038	[-0.57, 0.64]	0.900	1			
Primary outcome domain	Disability compared to Composite							
	Retrosp. vs. Prosp.	0.301	[-1.89, 2.49]	0.784	1	0.515	19.0%	(90)
	Nonreg. vs. Prosp.	-0.279	[-2.45, 1.90]	0.799	1			
	Pain compared to Composite							
	Retrosp. vs. Prosp.	-0.200	[-2.03, 1.63]	0.827	1			
	Nonreg. vs. Prosp.	0.154	[-1.82, 2.12]	0.876	1			
	PBF compared to Composite							
	Retrosp. vs. Prosp.	0.353	[-1.24, 1.95]	0.659	1			
	Nonreg. vs. Prosp.	-0.285	[-2.02, 1.45]	0.744	1			

Covariate	Comparison	Interaction	95% CI	P-value	Bonferroni-corrected P-value	Tau²	Tau² impact (% reduction of between study variance)	Studies excluded (missing variables)
Baseline measurement timing	Before surgery compared to after							
	Retrosp. vs. Prosp.	0.033	[-1.27, 1.34]	0.959	1	0.511	19.6%	(31,50,57,90)
	Nonreg. vs. Prosp.	0.051	[-1.18, 1.28]	0.934	1			
Time to follow-up	100 days longer							
	Retrosp. vs. Prosp.	0.196	[-0.06, 0.45]	0.135	1	0.457	28.1%	(16,90)
	Nonreg. vs. Prosp.	-0.060	[-0.21, 0.09]	0.416	1			

Appendix 7 Meta-regression interaction analysis. Primary outcome hierarchy levels: 1 = online registration, 2 = trial report, 3 = sample size calculation, 4,5 = emphases in abstract/objectives/results/discussion (grouped as there were too few), 6 = first outcome in results. PBF = Performance-based-function. Includes the 70 trials reporting extractable and comparable primary outcome measures, trials omitted from the analysis are referenced. Raw Tau² = 0.6258. References for excluded studies available in Appendix 10.
