# Supplementary material for: Registration and reporting characteristics of trials investigating exercise therapy following total knee arthroplasty: a systematic review
Source: Acta Orthop. 2026 Jun 22;97:408–16. doi: 10.2340/17453674.2026.46047 (PMC13284969; doi:10.2340/17453674.2026.46047)

Sensitivity analysis – Extended forest plot – trials recruiting before July 1st 2005 omitted.

Registration	Outcome hierarchy level	Estimate (95% CI)
**Prospective**		
Larsen (2024)	1	-0.30 (-0.77 : 0.2)
Buhagiar (2017)	1	-0.01 (-0.31 : 0.3)
Lenguerrand (2020)	1	0.14 (-0.16 : 0.4)
Hamilton (2020)	1	0.20 (-0.01 : 0.4)
Sattler (2019)	2	0.47 (-0.04 : 1.0)
Cheng (2022)	2	-0.17 (-0.87 : 0.5)
Fransen (2017)	1	-0.03 (-0.24 : 0.2)
Han (2015)	1	0.05 (-0.15 : 0.3)
Do and Yim (2020)	6	0.12 (-0.53 : 0.8)
**Retrospective**		
Bade (2017)	1	0.14 (-0.18 : 0.4)
Jakobsen (2014)	1	-0.29 (-0.75 : 0.2)
Christiansen (2015)	1	-0.28 (-1.05 : 0.5)
Harmer (2009)	4	-0.03 (-0.42 : 0.4)
Kelly (2015)	1	0.11 (-0.52 : 0.7)
Alsayani (2024)	6	0.55 (-0.14 : 1.2)
Carozzo (2022)	3	0.91 (0.27 : 1.6)
Trudelle-Jackson (2020)	3	-0.46 (-1.52 : 0.6)
Ko (2013)	2	-0.45 (-0.75 : -0.1)
Tanaka (2019)	6	-0.15 (-0.74 : 0.4)
Xu (2021)	6	-0.12 (-0.50 : 0.2)
Moffet (2015)	1	0.15 (-0.14 : 0.4)
Heikkilä (2017)	4	0.18 (-0.20 : 0.6)
Maeda (2024)	6	0.31 (-0.14 : 0.8)
Bruun-Olsen (2013)	1	0.64 (0.11 : 1.2)
Jacksteit (2021)	2	0.75 (0.15 : 1.4)
An (2023)	6	1.10 (0.44 : 1.8)
Lee (2021)	6	1.34 (0.65 : 2.0)
Husby (2018)	2	1.40 (0.66 : 2.1)
Moutzouri (2018)	2	1.99 (1.32 : 2.6)
Jiao (2023)	2	3.50 (2.79 : 4.2)
Cetinkaya (2022)	4	3.60 (2.78 : 4.4)
**Not registered**		
Chow (2010)	6	0.00 (-0.47 : 0.5)
den Hertog (2012)	2	0.15 (-0.18 : 0.5)
Zietek (2015)	6	0.24 (-0.25 : 0.7)
Schulz (2018)	6	0.52 (-0.03 : 1.1)
Mau-Moeller (2014)	2	0.81 (0.16 : 1.5)
Can (2023)	6	-0.37 (-1.01 : 0.3)
Levine (2013)	5	-0.15 (-0.67 : 0.4)
Yang (2013)	4	-0.12 (-0.90 : 0.6)
Dujin Park (2012)	4	-0.02 (-0.60 : 0.6)
Johnson (2010)	4	-0.01 (-0.94 : 0.9)
Pastore (2015)	6	0.00 (-0.69 : 0.7)
Eymir (2021)	3	0.06 (-0.31 : 0.4)
Shabbir (2017)	2	0.14 (-0.35 : 0.6)
Tousignant (2011)	6	0.26 (-0.34 : 0.9)
Park (2024)	6	0.45 (-0.69 : 1.6)
Tsukada (2020)	6	0.59 (-0.03 : 1.2)
Suh (2017)	4	0.62 (-0.06 : 1.3)
Hardt (2018)	3	0.70 (0.12 : 1.3)
Hepperger (2016)	6	1.00 (0.40 : 1.6)
Teissier (2020)	6	1.21 (0.29 : 2.1)
Bily (2016)	4	1.30 (0.73 : 1.9)
Unver (2016)	4	1.34 (0.78 : 1.9)
Liao (2013)	5	1.36 (0.95 : 1.8)
Papotto (2012)	3	1.42 (0.47 : 2.4)
Akbaba (2014)	4	1.51 (0.82 : 2.2)
Li (2019)	6	1.65 (1.21 : 2.1)
Molla (2017)	4	1.77 (1.05 : 2.5)
Karaman (2017)	3	2.04 (1.23 : 2.9)
Warner (2020)	2	3.61 (2.66 : 4.6)

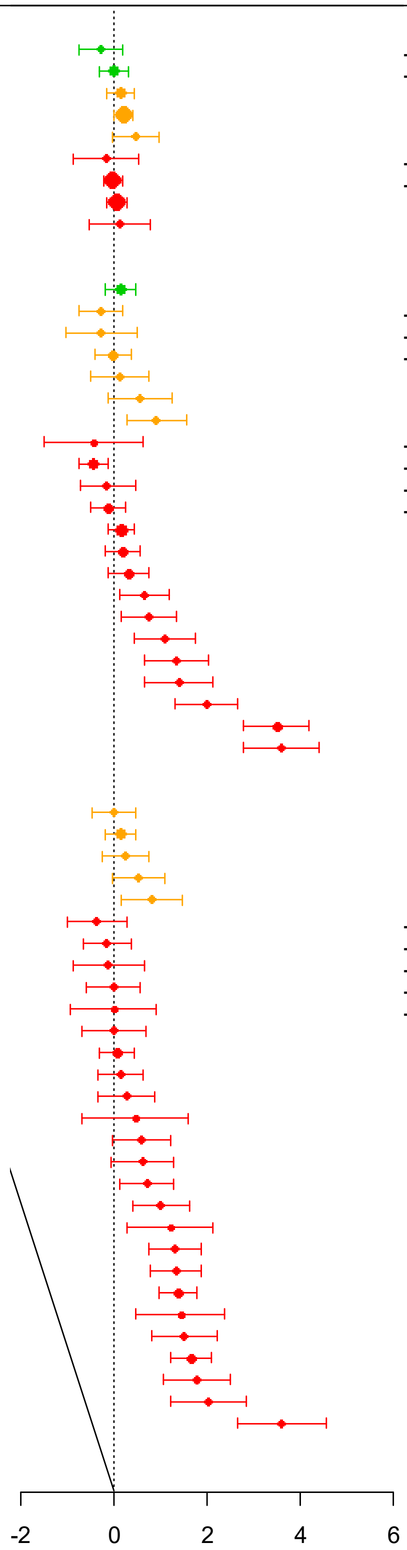

Sensitivity analysis – Funnel plot – trials recruiting before July 1st 2005 omitted.

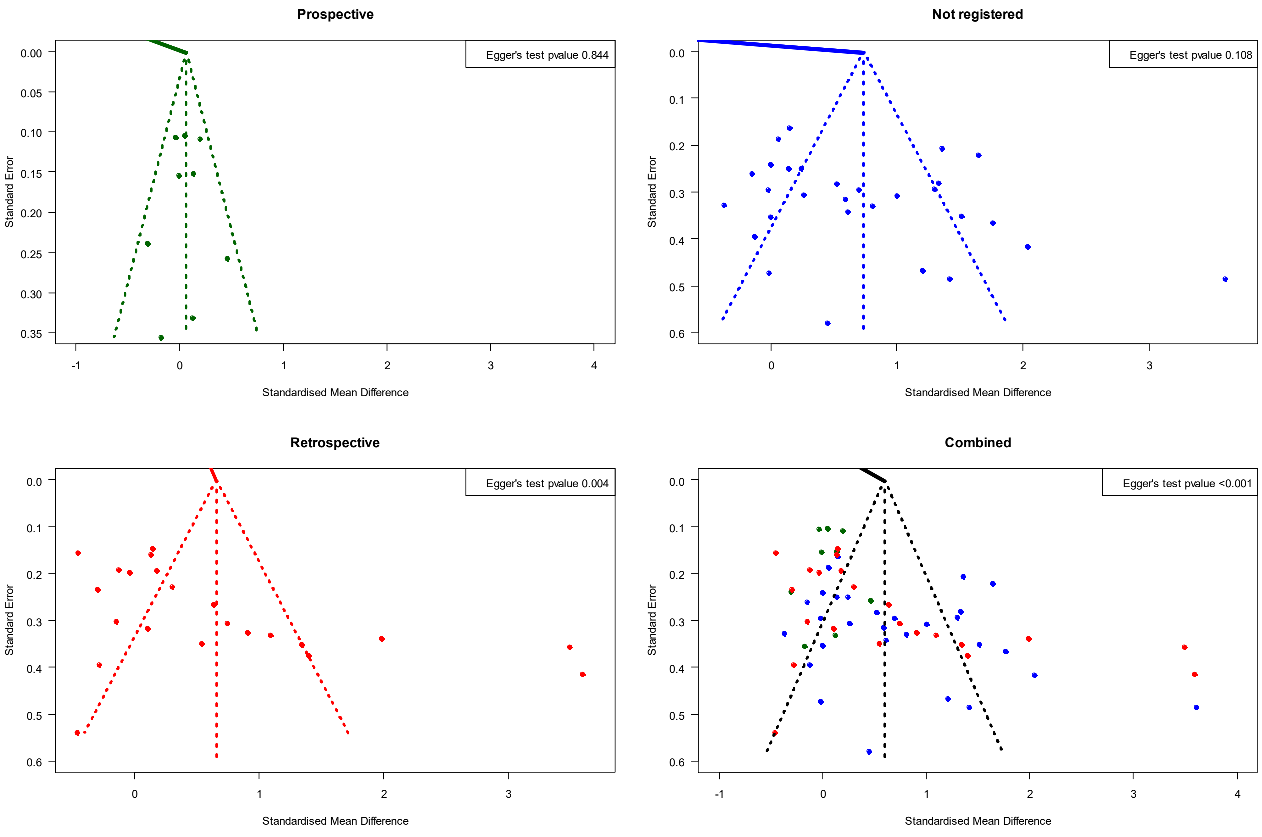

Sensitivity analysis – Combined forest plot – trials recruiting before July 1st 2005 omitted.

Registration	Number of studies	Number of participants	I^2	Estimate (95% CI)
Prospective	9	1568	0.01	0.06 (-0.03 : 0.16)
Retrospective	22	1549	0.91	0.66 (0.16 : 1.15)
Not registered	29	1498	0.84	0.74 (0.42 : 1.05)
Total	60	4615	0.88	0.60 (0.36 : 0.83)

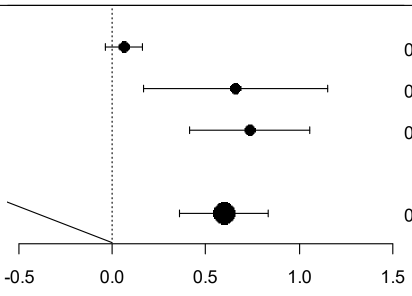

Supplement: Supplementary file 8 [file ActaO-97-46047-s8.pdf]
