# Supplementary material for: Registration and reporting characteristics of trials investigating exercise therapy following total knee arthroplasty: a systematic review
Source: Acta Orthop. 2026 Jun 22;97:408–16. doi: 10.2340/17453674.2026.46047 (PMC13284969; doi:10.2340/17453674.2026.46047)
Supplement: Supplementary file 9 [file ActaO-97-46047-s9.pdf]

Amendments since protocol registration

Available through registration on OSF, in original protocol document, and statistical analysis plan document. Since the original protocol was published, the following changes were made:

Amendment(s) to extracted outcome variables:

1st Amendment: Change from three extracted outcomes (pain, disability, performance-based function), using the hierarchy to using the hierarchy to select a single outcome.

Reason: As we always intended to compare the primary outcome in each trial across domains, it became redundant to also collect "secondary primary outcomes" in the other domains. We believed that sticking with a single outcome would make interpretations more focused on the methodological choices made in trials, the conciseness of their reporting, and its impact on the effect estimates.

Timing: This change was made after the search was originally completed, during May 2021, before data-extraction was initiated on June 1st, 2021.

2nd Amendment: Two data variables were dropped: publisher impact factor and external funding.

Reason: It turned out to be very problematic and time consuming to find verifiable and accurate impact factor values for the time the trials were published. External funding was omitted as it was considered outside the scope of the current review.

Timing: The variables were dropped shortly after data-extraction was initiated in 2020.

Amendment(s) to eligibility criteria since registration

Amendment: Trials investigating the effect of a non-exercise component, added to one of two groups with the same underlying exercise intervention were excluded from the review.

Reason: When the search was updated in 2024, during full-text assessment, it became clear that a large proportion of trials were investigating the effect of a non-exercise variable (such as drugs, massage,

behavior change, or continuous passive motion), with the same underlying exercise therapy in groups (e.g. comparing effects of 1) exercise therapy and massage against 2) exercise therapy alone). In the protocol research question was formulated with the intent of finding trials comparing different exercise modes.

Timing: This was implemented during title/abstract screening during the update of the search initiated on 12th of August 2024, with the change noted on 15th of November 2024.

Amendment(s) to analyses since registration:

1st Amendment: Grouping at three instead of two levels. Registration status was changed from Prospective- and Non-prospective registration, to Prospectively-, Retrospectively-, and Unregistered.

Reason: During the last search update, it was clear that several trials were not timely registered due to oversight, and that some these retrospectively registered trials were better reported than most non-registered trials. By presenting 3 layers of comparison, we hoped to enable a more nuanced representation of non-prospectively registered trials and clarify differences between trials with retrospective registration and no registration.

Timing: This was implemented, and described in the SAP, which was made available online on 6th of March 2025, before statistical analyses were undertaken.

2nd Amendment: The Chi-square test or Fishers exact test was planned for analyses of differences between categorical variables reporting study characteristics, but none were conducted.

Reason: The descriptive table 1 is intended to be descriptive and provide context, therefore statistical testing for significant differences may be misleading.

Timing: Implemented after the SAP was registered on 6th of March 2025, but before data analysis was initiated.
