# Supplementary material for: Registration and reporting characteristics of trials investigating exercise therapy following total knee arthroplasty: a systematic review
Source: Acta Orthop. 2026 Jun 22;97:408–16. doi: 10.2340/17453674.2026.46047 (PMC13284969; doi:10.2340/17453674.2026.46047)
Supplement: Supplementary file 10 [file ActaO-97-46047-s10.pdf]

Trials included in systematic review (n=94):

(1–94)

Trials presenting data suitable for analyses (n=70):

(1–3,5,6,10–13,15–18,21,22,26,28,30–38,40–44,46–54,56–58,60–63,65,67–70,72,73,76,78–81,83–88,90–94)

Trial reporting differences section – with trial references:

"In the 13 prospectively registered trials: A single primary outcome consistent with a single prospectively registered, unaltered, primary outcome was reported in 4 of the 13 (30.8%) trials (8,30,49,51)*. The primary outcome was changed or switched in 2 of the 13 (15.4%) trials* (11,15)*. Multiple primary outcomes were registered and/or reported in 7 of the 13 (53.9%) trials* (22,28,31,39,55,76,77)*."*

References:

1. Akbaba YA, Yeldan I, Guney N, Ozdincler AR. Intensive supervision of rehabilitation programme improves balance and functionality in the short term after bilateral total knee arthroplasty. Knee Surg Sports Traumatol Arthrosc. 2016 Jan;24(1):26–33. doi:10.1007/s00167-014-3179-y

2. Alsayani KYA, Baş Aslan U, Bayrak G, Şavkın R, Büker N, Güngör HR. Comparison of the effectiveness of late-phase clinic-based and home-based progressive resistance training in female patients with total knee arthroplasty. Physiotherapy Theory and Practice. 2024 Aug 2;40(8):1687–98. doi:10.1080/09593985.2023.2205925

3. An J, Son YW, Lee BH. Effect of Combined Kinematic Chain Exercise on Physical Function, Balance Ability, and Gait in Patients with Total Knee Arthroplasty: A Single-Blind Randomized Controlled Trial. IJERPH. 2023 Feb 16;20(4):3524. doi:10.3390/ijerph20043524

4. Bäcker HC, Wu CH, Schulz MRG, Weber-Spickschen TS, Perka C, Hardt S. App-based rehabilitation program after total knee arthroplasty: a randomized controlled trial. Arch Orthop Trauma Surg. 2021 Sep;141(9):1575–82. doi:10.1007/s00402-021-03789-0

5. Bade MJ, Struessel T, Dayton M, Foran J, Kim RH, Miner T, et al. Early High-Intensity Versus Low-Intensity Rehabilitation After Total Knee Arthroplasty: A Randomized Controlled Trial. Arthritis Care & Research. 2017 Sep;69(9):1360–8. doi:10.1002/acr.23139

6. Bily W, Franz C, Trimmel L, Loefler S, Cvecka J, Zampieri S, et al. Effects of Leg-Press Training With Moderate Vibration on Muscle Strength, Pain, and Function After Total Knee Arthroplasty: A Randomized Controlled Trial. Archives of Physical Medicine and Rehabilitation. 2016 Jun;97(6):857–65. doi:10.1016/j.apmr.2015.12.015

7. Bini S, Mahajan J. Clinical outcomes of remote asynchronous telerehabilitation are equivalent to traditional therapy following total knee arthroplasty: A randomized control study. J Telemed Telecare. 2017 Feb;23(2):239–47. doi:10.1177/1357633X16634518

8. Bohl DD, Li J, Calkins TE, Darrith B, Edmiston TA, Nam D, et al. Physical Therapy on Postoperative Day Zero Following Total Knee Arthroplasty: A Randomized, Controlled Trial of 394 Patients. The Journal of Arthroplasty. 2019 Jul;34(7):S173-S177.e1. doi:10.1016/j.arth.2019.02.010

9. Bradbury TL, McConnell MJ, Whitacre D, Naylor BH, Gibson BT, DeCook CA. A Remote Physical Therapy Program Demonstrates Similar Outcomes Compared to In-Person, Supervised Physical Therapy After Same-Day Discharge Total Knee Arthroplasty: A Randomized Clinical Trial. The Journal of Arthroplasty. 2024 Nov;39(11):2725-2730.e4. doi:10.1016/j.arth.2024.05.040

10. Bruun-Olsen V, Heiberg KE, Wahl AK, Mengshoel AM. The immediate and long-term effects of a walking-skill program compared to usual physiotherapy care in patients who have undergone total knee arthroplasty (TKA): a randomized controlled trial. Disability and Rehabilitation. 2013 Nov;35(23):2008–15. doi:10.3109/09638288.2013.770084

11. Buhagiar MA, Naylor JM, Harris IA, Xuan W, Kohler F, Wright R, et al. Effect of Inpatient Rehabilitation vs a Monitored Home-Based Program on Mobility in Patients With Total Knee Arthroplasty: The HIHO Randomized Clinical Trial. JAMA. 2017 Mar 14;317(10):1037. doi:10.1001/jama.2017.1224

12. Carozzo S, Vatrano M, Coschignano F, Battaglia R, Calabrò RS, Pignolo L, et al. Efficacy of Visual Feedback Training for Motor Recovery in Post-Operative Subjects with Knee Replacement: A Randomized Controlled Trial. JCM. 2022 Dec 11;11(24):7355. doi:10.3390/jcm11247355

13. Çetinkaya F, Karakoyun A. The effects of elastic band exercise on the pain, kinesiophobia, functional, and psychological status after total knee arthroplasty: a randomized controlled trial. Clin Rheumatol. 2022 Oct;41(10):3179–88. doi:10.1007/s10067-022-06266-0

14. Chang HL, Hsu MF, Wong TH, Chung YC, Huang HL. Effects of a Hybrid Teaching Program on Lower Limb Muscle Strength, Knee Function, and Depression in Older Adults After Total Knee Replacement: A Randomized Controlled Trial. Research in Gerontological Nursing. 2024 Jan;17(1):31–40. doi:10.3928/19404921-20230918-01

15. Cheng YY, Liu CC, Lin SY, Lee CH, Chang ST, Wang SP. Comparison of the Therapeutic Effects Between Isokinetic and Isotonic Strength Training in Patients After Total Knee Replacement: A Prospective, Randomized Controlled Trial. Orthopaedic Journal of Sports Medicine. 2022 Jun 1;10(6):23259671221105852. doi:10.1177/23259671221105852

16. Chow TP, Ng GY. Active, passive and proprioceptive neuromuscular facilitation stretching are comparable in improving the knee flexion range in people with total knee replacement: a randomized controlled trial. Clin Rehabil. 2010 Oct;24(10):911–8. doi:10.1177/0269215510367992

17. Christiansen CL, Bade MJ, Davidson BS, Dayton MR, Stevens-Lapsley JE. Effects of Weight-Bearing Biofeedback Training on Functional Movement Patterns Following Total Knee Arthroplasty: A Randomized Controlled Trial. Journal of Orthopaedic & Sports Physical Therapy. 2015 Sep;45(9):647–55. doi:10.2519/jospt.2015.5593

18. Codine Ph, Dellemme Y, Denis-Laroque F, Herisson Ch. The use of low velocity submaximal eccentric contractions of the hamstring for recovery of full extension after total knee replacement: A

randomized controlled study. Isokinetics and Exercise Science. 2004 Aug;12(3):215–8. doi:10.3233/IES-2004-0177

19. Crawford DA, Duwelius PJ, Sneller MA, Morris MJ, Hurst JM, Berend KR, et al. 2021 Mark Coventry Award: Use of a smartphone-based care platform after primary partial and total knee arthroplasty: a prospective randomized controlled trial. The Bone & Joint Journal. 2021 Jun 1;103-B(6 Supple A):3–12. doi:10.1302/0301-620X.103B6.BJJ-2020-2352.R1

20. DeJong G, Hsieh CJ, Vita MT, Zeymo A, Boucher HR, Thakkar SC. Innovative Devices Did Not Provide Superior Total Knee Arthroplasty Outcomes in Post-Operative Rehabilitation: Results From a Four-Arm Randomized Clinical Trial. The Journal of Arthroplasty. 2020 Aug;35(8):2054–65. doi:10.1016/j.arth.2020.03.048

21. Den Hertog A, Gliesche K, Timm J, Mühlbauer B, Zebrowski S. Pathway-controlled fast-track rehabilitation after total knee arthroplasty: a randomized prospective clinical study evaluating the recovery pattern, drug consumption, and length of stay. Arch Orthop Trauma Surg. 2012 Aug;132(8):1153–63. doi:10.1007/s00402-012-1528-1

22. Do K, Yim J. Effects of Muscle Strengthening around the Hip on Pain, Physical Function, and Gait in Elderly Patients with Total Knee Arthroplasty: A Randomized Controlled Trial. Healthcare. 2020 Nov 17;8(4):489. doi:10.3390/healthcare8040489

23. Doerfler D, Gurney B, Mermier C, Rauh M, Black L, Andrews R. High-Velocity Quadriceps Exercises Compared to Slow-Velocity Quadriceps Exercises Following Total Knee Arthroplasty: A Randomized Clinical Study. Journal of Geriatric Physical Therapy. 2016 Oct;39(4):147–58. doi:10.1519/JPT.0000000000000071

24. Eisermann U, Haase I, Kladny B. Computer-Aided Multimedia Training in Orthopedic Rehabilitation: American Journal of Physical Medicine & Rehabilitation. 2004 Sep;83(9):670–80. doi:10.1097/01.PHM.0000137307.44173.5D

25. Evgeniadis G, Beneka A, Malliou P, Mavromoustakos S, Godolias G. Effects of pre- or postoperative therapeutic exercise on the quality of life, before and after total knee arthroplasty for osteoarthritis. BMR. 2008 Sep 1;21(3):161–9. doi:10.3233/BMR-2008-21303

26. Eymir M, Erduran M, Ünver B. Active heel-slide exercise therapy facilitates the functional and proprioceptive enhancement following total knee arthroplasty compared to continuous passive motion. Knee Surg Sports Traumatol Arthrosc. 2021 Oct;29(10):3352–60. doi:10.1007/s00167-020-06181-4

27. Fleischman AN, Crizer MP, Tarabichi M, Smith S, Rothman RH, Lonner JH, et al. 2018 John N. Insall Award: Recovery of Knee Flexion With Unsupervised Home Exercise Is Not Inferior to Outpatient Physical Therapy After TKA: A Randomized Trial. Clin Orthop Relat Res. 2019 Jan;477(1):60–9. doi:10.1097/CORR.0000000000000561

28. Fransen M, Nairn L, Bridgett L, Crosbie J, March L, Parker D, et al. Post-Acute Rehabilitation After Total Knee Replacement: A Multicenter Randomized Clinical Trial Comparing Long-Term Outcomes. Arthritis Care & Research. 2017 Feb;69(2):192–200. doi:10.1002/acr.23117

29. Fung V, Ho A, Shaffer J, Chung E, Gomez M. Use of Nintendo Wii Fit™ in the rehabilitation of outpatients following total knee replacement: a preliminary randomised controlled trial. Physiotherapy. 2012 Sep;98(3):183–8. doi:10.1016/j.physio.2012.04.001

30. Hamilton DF, Beard DJ, Barker KL, Macfarlane GJ, Tuck CE, Stoddart A, et al. Targeting rehabilitation to improve outcomes after total knee arthroplasty in patients at risk of poor outcomes: randomised controlled trial. BMJ. 2020 Oct 13;m3576. doi:10.1136/bmj.m3576

31. Han ASY, Nairn L, Harmer AR, Crosbie J, March L, Parker D, et al. Early Rehabilitation After Total Knee Replacement Surgery: A Multicenter, Noninferiority, Randomized Clinical Trial Comparing a Home Exercise Program With Usual Outpatient Care. Arthritis Care & Research. 2015 Feb;67(2):196–202. doi:10.1002/acr.22457

32. Hardt S, Schulz MRG, Pfitzner T, Wassilew G, Horstmann H, Liodakis E, et al. Improved early outcome after TKA through an app-based active muscle training programme—a randomized-controlled trial. Knee Surg Sports Traumatol Arthrosc. 2018 Nov;26(11):3429–37. doi:10.1007/s00167-018-4918-2

33. Harmer AR, Naylor JM, Crosbie J, Russell T. Land-based versus water-based rehabilitation following total knee replacement: A randomized, single-blind trial. Arthritis & Rheumatism. 2009 Feb 15;61(2):184–91. doi:10.1002/art.24420

34. Heikkilä A, Sevander-Kreus N, Häkkinen A, Vuorenmaa M, Salo P, Konsta P, et al. Effect of total knee replacement surgery and postoperative 12 month home exercise program on gait parameters. Gait & Posture. 2017 Mar;53:92–7. doi:10.1016/j.gaitpost.2017.01.004

35. Hepperger C, Gföller P, Hoser C, Ulmer H, Fischer F, Schobersberger W, et al. The effects of a 3-month controlled hiking programme on the functional abilities of patients following total knee arthroplasty: a prospective, randomized trial. Knee Surg Sports Traumatol Arthrosc. 2017 Nov;25(11):3387–95. doi:10.1007/s00167-016-4299-3

36. Husby VS, Foss OA, Husby OS, Winther SB. Randomized controlled trial of maximal strength training vs. standard rehabilitation following total knee arthroplasty. Eur J Phys Rehabil Med. 2018 Jun;54(3). doi:10.23736/S1973-9087.17.04712-8

37. Jacksteit R, Stöckel T, Behrens M, Feldhege F, Bergschmidt P, Bader R, et al. Low-Load Unilateral and Bilateral Resistance Training to Restore Lower Limb Function in the Early Rehabilitation After Total Knee Arthroplasty: A Randomized Active-Controlled Clinical Trial. Front Med. 2021 Jun 22;8:628021. doi:10.3389/fmed.2021.628021

38. Jakobsen TL, Kehlet H, Husted H, Petersen J, Bandholm T. Early Progressive Strength Training to Enhance Recovery After Fast-Track Total Knee Arthroplasty: A Randomized Controlled Trial. Arthritis Care & Research. 2014 Dec;66(12):1856–66. doi:10.1002/acr.22405

39. Janhunen M, Katajapuu N, Paloneva J, Pamilo K, Oksanen A, Keemu H, et al. Effects of a home-based, exergaming intervention on physical function and pain after total knee replacement in older adults: a randomised controlled trial. BMJ Open Sport Exerc Med. 2023 Mar;9(1):e001416. doi:10.1136/bmjsem-2022-001416

40. Jiao S, Feng Z, Dai T, Huang J, Liu R, Meng Q. High-Intensity Progressive Rehabilitation Versus Routine Rehabilitation After Total Knee Arthroplasty: A Randomized Controlled Trial. The Journal of Arthroplasty. 2024 Mar;39(3):665-671.e2. doi:10.1016/j.arth.2023.08.052

41. Johnson AW, Myrer JW, Hunter I, Feland JB, Hopkins JT, Draper DO, et al. Whole-body vibration strengthening compared to traditional strengthening during physical therapy in individuals with total knee arthroplasty. Physiotherapy Theory and Practice. 2010 Jan;26(4):215–25. doi:10.3109/09593980902967196

42. Karaman A, Yuksel I, Kinikli GI, Caglar O. Do Pilates-based exercises following total knee arthroplasty improve postural control and quality of life? Physiotherapy Theory and Practice. 2017 Apr 3;33(4):289–95. doi:10.1080/09593985.2017.1289578

43. Kauppila AM, Kyllönen E, Ohtonen P, Hämäläinen M, Mikkonen P, Laine V, et al. Multidisciplinary rehabilitation after primary total knee arthroplasty: a randomized controlled study of its effects on functional capacity and quality of life. Clin Rehabil. 2010 May;24(5):398–411. doi:10.1177/0269215509346089

44. Kelly MA, Finley M, Lichtman SW, Hyland MR, Edeer AO. Comparative Analysis of High-Velocity Versus Low-Velocity Exercise on Outcomes After Total Knee Arthroplasty: A Randomized Clinical Trial. Journal of Geriatric Physical Therapy. 2016 Oct;39(4):178–89. doi:10.1519/JPT.0000000000000070

45. Khanli MM, Akbari M, Amiri A. The Effect of Early Hip-strengthening on Physical Function in Patients With Unilateral Total Knee Arthroplasty. J Arak Uni Med Sci. 2021 Feb 1;23(6):912–25. doi:10.32598/jams.23.6.6045.1

46. Kiraç Can E, Tomruk M, Gelecek N. BİLATERAL TOTAL DİZ PROTEZİ SONRASI ERKEN İLERLEYİCİ KAPALI KİNETİK ZİNCİR EGZERSİZLERİNİN STANDART EGZERSİZ PROGRAMINA GÖRE ETKİLERİ - RANDOMİZE KONTROLLÜ ÇALIŞMA. Türk Fizyoterapi ve Rehabilitasyon Dergisi. 2023 Apr 20;34(1):102–14. doi:10.21653/tjpr.1056788

47. Ko V, Naylor J, Harris I, Crosbie J, Yeo A, Mittal R. One-to-One Therapy Is Not Superior to Group or Home-Based Therapy After Total Knee Arthroplasty: A Randomized, Superiority Trial. Journal of Bone and Joint Surgery. 2013 Nov 6;95(21):1942–9. doi:10.2106/JBJS.L.00964

48. Kramer JF, Speechley M, Bourne R, Rorabeck C, Vaz M. Comparison of Clinic- and Home-Based Rehabilitation Programs After Total Knee Arthroplasty. Clinical Orthopaedics & Related Research. 2003 May;410:225–34. doi:10.1097/01.blo.0000063600.67412.11

49. Larsen JB, Skou ST, Laursen M, Bruun NH, Arendt-Nielsen L, Madeleine P. Exercise and Pain Neuroscience Education for Patients With Chronic Pain After Total Knee Arthroplasty: A Randomized Clinical Trial. JAMA Netw Open. 2024 May 24;7(5):e2412179. doi:10.1001/jamanetworkopen.2024.12179

50. Lee HG, An J, Lee BH. The Effect of Progressive Dynamic Balance Training on Physical Function, The Ability to Balance and Quality of Life Among Elderly Women Who Underwent a Total Knee Arthroplasty: A Double-Blind Randomized Control Trial. IJERPH. 2021 Mar 3;18(5):2513. doi:10.3390/ijerph18052513

51. Lenguerrand E, Artz N, Marques E, Sanderson E, Lewis K, Murray J, et al. Effect of Group-Based Outpatient Physical Therapy on Function After Total Knee Replacement: Results From a Multicenter Randomized Controlled Trial. Arthritis Care & Research. 2020 Jun;72(6):768–77. doi:10.1002/acr.23909

52. Lenssen AF, Crijns YH, Waltjé EM, Van Steyn MJ, Geesink RJ, Van Den Brandt PA, et al. Efficiency of immediate postoperative inpatient physical therapy following total knee arthroplasty: an RCT. BMC Musculoskelet Disord. 2006 Dec;7(1):71. doi:10.1186/1471-2474-7-71

53. Levine M, McElroy K, Stakich V, Cicco J. Comparing Conventional Physical Therapy Rehabilitation With Neuromuscular Electrical Stimulation After TKA. Orthopedics. 2013 Mar;36(3). doi:10.3928/01477447-20130222-20

54. Li L, Cheng S, Wang G, Duan G, Zhang Y. Tai chi chuan exercises improve functional outcomes and quality of life in patients with primary total knee arthroplasty due to knee osteoarthritis. Complementary Therapies in Clinical Practice. 2019 May;35:121–5. doi:10.1016/j.ctcp.2019.02.003

55. Liao CD, Chiu YS, Ku JW, Huang SW, Liou TH. Effects of Elastic Resistance Exercise on Postoperative Outcomes Linked to the ICF Core Sets for Osteoarthritis after Total Knee Replacement in Overweight and Obese Older Women with Sarcopenia Risk: A Randomized Controlled Trial. JCM. 2020 Jul 11;9(7):2194. doi:10.3390/jcm9072194

56. Liao CD, Liou TH, Huang YY, Huang YC. Effects of balance training on functional outcome after total knee replacement in patients with knee osteoarthritis: a randomized controlled trial. Clin Rehabil. 2013 Aug;27(8):697–709. doi:10.1177/0269215513476722

57. Liebs TR, Herzberg W, Rüther W, Haasters J, Russlies M, Hassenpflug J. Ergometer Cycling After Hip or Knee Replacement Surgery: A Randomized Controlled Trial. The Journal of Bone and Joint Surgery-American Volume. 2010 Apr;92(4):814–22. doi:10.2106/JBJS.H.01359

58. Liebs TR, Herzberg W, Rüther W, Haasters J, Russlies M, Hassenpflug J. Multicenter Randomized Controlled Trial Comparing Early Versus Late Aquatic Therapy After Total Hip or Knee Arthroplasty. Archives of Physical Medicine and Rehabilitation. 2012 Feb;93(2):192–9. doi:10.1016/j.apmr.2011.09.011

59. Madsen M, Larsen K, Madsen IK, Søe H, Hansen TB. Late group-based rehabilitation has no advantages compared with supervised home-exercises after total knee arthroplasty. 2013 Apr;(60(4):A4607).

60. Maeda T, Sasaki E, Kasai T, Igarashi S, Wakai Y, Sasaki T, et al. Therapeutic effect of knee extension exercise with single-joint hybrid assistive limb following total knee arthroplasty: a prospective, randomized controlled trial. Sci Rep. 2024 Feb 16;14(1):3889. doi:10.1038/s41598-024-53891-7

61. Mau-Moeller A, Behrens M, Finze S, Bruhn S, Bader R, Mittelmeier W. The effect of continuous passive motion and sling exercise training on clinical and functional outcomes following total knee arthroplasty: a randomized active-controlled clinical study. Health Qual Life Outcomes. 2014;12(1):68. doi:10.1186/1477-7525-12-68

62. Moffet H, Collet JP, Shapiro SH, Paradis G, Marquis F, Roy L. Effectiveness of intensive rehabilitation on functional ability and quality of life after first total knee arthroplasty: a single-blind randomized controlled trial. Archives of Physical Medicine and Rehabilitation. 2004 Apr;85(4):546–56. doi:10.1016/j.apmr.2003.08.080

63. Moffet H, Tousignant M, Nadeau S, Mérette C, Boissy P, Corriveau H, et al. In-Home Telerehabilitation Compared with Face-to-Face Rehabilitation After Total Knee Arthroplasty: A Noninferiority Randomized Controlled Trial. The Journal of Bone and Joint Surgery-American Volume. 2015 Jul;97(14):1129–41. doi:10.2106/JBJS.N.01066

64. Monticone M, Ferrante S, Rocca B, Salvaderi S, Fiorentini R, Restelli M, et al. Home-Based Functional Exercises Aimed at Managing Kinesiophobia Contribute to Improving Disability and Quality of Life of Patients Undergoing Total Knee Arthroplasty: A Randomized Controlled Trial. Archives of Physical Medicine and Rehabilitation. 2013 Feb;94(2):231–9. doi:10.1016/j.apmr.2012.10.003

65. Moutzouri M, Gleeson N, Coutts F, Tsepis E, Gliatis J. Early self-managed focal sensorimotor rehabilitative training enhances functional mobility and sensorimotor function in patients following total

knee replacement: a controlled clinical trial. Clin Rehabil. 2018 Jul;32(7):888–98. doi:10.1177/0269215518757291 PubMed PMID: 29473481.

66. Núñez-Cortés R, López-Bueno L, López-Bueno R, Cuenca-Martínez F, Suso-Martí L, Silvestre A, et al. Acute Effects of In-Hospital Resistance Training on Clinical Outcomes in Patients Undergoing Total Knee Arthroplasty: A Randomized Controlled Trial. Am J Phys Med Rehabil. 2024 May;103(5):401–9. doi:10.1097/PHM.0000000000002366

67. Papotto BA, Mills T. Treatment of Severe Flexion Deficits Following Total Knee Arthroplasty: A Randomized Clinical Trial. Orthopaedic Nursing. 2012 Jan;31(1):29–34. doi:10.1097/NOR.0b013e3182419662

68. Park D, Kim J, Lee H. Effectiveness of Modified Quadriceps Femoris Muscle Setting Exercise for the Elderly in Early Rehabilitation after Total Knee Arthroplasty. J Phys Ther Sci. 2012;24(1):27–30. doi:10.1589/jpts.24.27

69. Park KJ, Seo TB, Kim YP. Effects of proprioceptive neuromuscular facilitation and both sides up ball exercise on pain level, range of motion, muscle function after total knee arthroplasty. J Exerc Rehabil. 2024 Feb 21;20(1):17–23. doi:10.12965/jer.2448004.002

70. Pastore E Silva AL. Estudo comparativo entre dois métodos de reabilitação fisioterapêutica na artroplastia total do joelho: protocolo padrão do IOT vs. protocolo avançado. Fisioter Bras. 2016 Jun 30;16(2):100–6. doi:10.33233/fb.v16i2.275

71. Piqueras M, Marco E, Coll M, Escalada F, Ballester A, Cinca C, et al. Effectiveness of an interactive virtual telerehabilitation system in patients after total knee arthoplasty: A randomized controlled trial. J Rehabil Med. 2013;45(4):392–6. doi:10.2340/16501977-1119

72. Rahmann AE, Brauer SG, Nitz JC. A Specific Inpatient Aquatic Physiotherapy Program Improves Strength After Total Hip or Knee Replacement Surgery: A Randomized Controlled Trial. Archives of Physical Medicine and Rehabilitation. 2009 May;90(5):745–55. doi:10.1016/j.apmr.2008.12.011

73. Rajan R, Pack Y, Jackson H, Gillies C, Asirvatham R. No need for outpatient physiotherapy following total knee arthroplastyA randomized trial of 120 patients. Acta Orthopaedica Scandinavica. 2004 Jan;75(1):71–3. doi:10.1080/00016470410001708140

74. Russell TG, Buttrum P, Wootton R, Jull GA. Low-bandwidth telerehabilitation for patients who have undergone total knee replacement: Preliminary results. J Telemed Telecare. 2003 Dec;9(2_suppl):44–7. doi:10.1258/135763303322596246

75. Russell TG, Buttrum P, Wootton R, Jull GA. Internet-Based Outpatient Telerehabilitation for Patients Following Total Knee Arthroplasty: A Randomized Controlled Trial. The Journal of Bone and Joint Surgery-American Volume. 2011 Jan;93(2):113–20. doi:10.2106/JBJS.I.01375

76. Sattler LN, Hing WA, Vertullo CJ. Pedaling-Based Protocol Superior to a 10-Exercise, Non-Pedaling Protocol for Postoperative Rehabilitation After Total Knee Replacement: A Randomized Controlled Trial. The Journal of Bone and Joint Surgery. 2019 Apr 17;101(8):688–95. doi:10.2106/JBJS.18.00898

77. Schache MB, McClelland JA, Webster KE. Incorporating hip abductor strengthening exercises into a rehabilitation program did not improve outcomes in people following total knee arthroplasty: a randomised trial. Journal of Physiotherapy. 2019 Jul;65(3):136–43. doi:10.1016/j.jphys.2019.05.008

78. Schulz M, Krohne B, Röder W, Sander K. Randomized, prospective, monocentric study to compare the outcome of continuous passive motion and controlled active motion after total knee arthroplasty. THC. 2018 Jun 29;26(3):499–506. doi:10.3233/THC-170850

79. Shabbir M, Umar B, Ehsan S, Munir S, Bunin U, Sarfraz K. Comparison of functional training and strength training in improving knee extension lag after first four weeks of total knee replacement [Internet]. 2017 [cited 2025 May 15]. Available from: https://www.biomedres.info/abstract/comparison-of-functional-training-and-strength-training-in-improving-knee-extension-lag-after-first-four-weeks-of-total-knee-repla-7757.html

80. Suh MJ, Kim BR, Kim SR, Han EY, Lee SY. Effects of Early Combined Eccentric-Concentric Versus Concentric Resistance Training Following Total Knee Arthroplasty. Ann Rehabil Med. 2017;41(5):816. doi:10.5535/arm.2017.41.5.816

81. Tanaka R, Hayashizaki T, Taniguchi R, Kobayashi J, Umehara T. Effect of an intensive functional rehabilitation program on the recovery of activities of daily living after total knee arthroplasty: A multicenter, randomized, controlled trial. Journal of Orthopaedic Science. 2020 Mar;25(2):285–90. doi:10.1016/j.jos.2019.04.009

82. Tanaka Y, Oka H, Nakayama S, Ueno T, Matsudaira K, Miura T, et al. Improvement of walking ability during postoperative rehabilitation with the hybrid assistive limb after total knee arthroplasty: A randomized controlled study. SAGE Open Medicine. 2017 Jan 1;5:2050312117712888. doi:10.1177/2050312117712888

83. Teissier V, Leclercq R, Schiano-Lomoriello S, Nizard R, Portier H. Does eccentric-concentric resistance training improve early functional outcomes compared to concentric resistance training after total knee arthroplasty? Gait & Posture. 2020 Jun;79:145–51. doi:10.1016/j.gaitpost.2020.04.020

84. Tousignant M, Moffet H, Boissy P, Corriveau H, Cabana F, Marquis F. A randomized controlled trial of home telerehabilitation for post-knee arthroplasty. J Telemed Telecare. 2011 Jun;17(4):195–8. doi:10.1258/jtt.2010.100602

85. Trudelle-Jackson E, Hines E, Medley A, Thompson M. Exploration of Habitual Walking Behavior and Home-Based Muscle Power Training in Individuals With Total Knee Arthroplasty. Journal of Physical Activity and Health. 2020 Mar 1;17(3):331–8. doi:10.1123/jpah.2019-0233

86. Tsukada Y, Matsuse H, Shinozaki N, Takano Y, Nago T, Shiba N. Combined Application of Electrically Stimulated Antagonist Muscle Contraction and Volitional Muscle Contraction Prevents Muscle Strength Weakness and Promotes Physical Function Recovery After Total Knee Arthroplasty: A Randomized Controlled Trial. Kurume Med J. 2018 Dec 31;65(4):145–54. doi:10.2739/kurumemedj.MS654007

87. Unver B, Bakirhan S, Karatosun V. Does a weight-training exercise programme given to patients four or more years after total knee arthroplasty improve mobility: A randomized controlled trial. Archives of Gerontology and Geriatrics. 2016 May;64:45–50. doi:10.1016/j.archger.2016.01.003

88. Valtonen A, Pöyhönen T, Sipilä S, Heinonen A. Effects of Aquatic Resistance Training on Mobility Limitation and Lower-Limb Impairments After Knee Replacement. Archives of Physical Medicine and Rehabilitation. 2010 Jun;91(6):833–9. doi:10.1016/j.apmr.2010.03.002

89. Vuorenmaa M, Ylinen J, Piitulainen K, Salo P, Kautiainen H, Pesola M, et al. Efficacy of a 12-month, monitored home exercise programme compared with normal care commencing 2 months after total knee

arthroplasty: A randomized controlled trial. J Rehabil Med. 2014;46(2):166–72. doi:10.2340/16501977-1242

90. Warner S, Ahmad A, Afzal MW, Khan S, Aslam MM, Gillani SA. Comparison of routine physical therapy exercises with and without core stability exercises in total knee replacement patients. Rawal Medical Journal. 2020 Dec 17;45(4):842–842.

91. Xu T, Yang D, Liu K, Gao Q, Lu H, Qiao Y, et al. Efficacy and safety of a self-developed home-based enhanced knee flexion exercise program compared with standard supervised physiotherapy to improve mobility and quality of life after total knee arthroplasty: a randomized control study. J Orthop Surg Res. 2021 Dec;16(1):382. doi:10.1186/s13018-021-02516-0

92. Yang TH, Yeh WL, Chen HY, Chen YF, Ni KC, Lee KH. Compare the Traditional Chinese Medicine Manipulation With Rehabilitation on In-Patients After Total Knee Arthroplasty. The Journal of Arthroplasty. 2013 Jun;28(6):954–9. doi:10.1016/j.arth.2012.07.045

93. Yousefian Molla R, Sadeghi H, Kahlaee AH. The Effect of Early Progressive Resistive Exercise Therapy on Balance Control of Patients With Total Knee Arthroplasty: A Randomized Controlled Trial. Topics in Geriatric Rehabilitation. 2017 Oct;33(4):286–94. doi:10.1097/TGR.0000000000000165

94. Zietek P, Zietek J, Szczypior K, Safranow K. Effect of adding one 15-minute-walk on the day of surgery to fast-track rehabilitation after total knee arthroplasty: a randomized, single-blind study. Eur J Phys Rehabil Med. 2015 Jun;51(3):245–52. PubMed PMID: 25230888.
